# Modeling of interaction between cytochrome c and the WD domains of Apaf-1: bifurcated salt bridges underlying apoptosome assembly

**DOI:** 10.1186/s13062-015-0059-4

**Published:** 2015-05-27

**Authors:** Daria N. Shalaeva, Daria V. Dibrova, Michael Y. Galperin, Armen Y. Mulkidjanian

**Affiliations:** School of Physics, Osnabrück University, 49069 Osnabrück, Germany; School of Bioengineering and Bioinformatics, 117999 Moscow, Russia; A.N. Belozersky Institute of Physico-Chemical Biology, Lomonosov Moscow State University, 117999 Moscow, Russia; National Center for Biotechnology Information, National Library of Medicine, National Institutes of Health, 20894 Bethesda, MD USA

**Keywords:** Apoptosis, WD40 domains, Hydrogen bond, Salt bridge, Apoptosis, Protein-protein interactions, Caspase, Molecular dynamics simulations, Sequence analysis, Evolution

## Abstract

**Background:**

Binding of cytochrome *c*, released from the damaged mitochondria, to the apoptotic protease activating factor 1 (Apaf-1) is a key event in the apoptotic signaling cascade. The binding triggers a major domain rearrangement in Apaf-1, which leads to oligomerization of Apaf-1/cytochrome *c* complexes into an apoptosome. Despite the availability of crystal structures of cytochrome *c* and Apaf-1 and cryo-electron microscopy models of the entire apoptosome, the binding mode of cytochrome *c* to Apaf-1, as well as the nature of the amino acid residues of Apaf-1 involved remain obscure.

**Results:**

We investigated the interaction between cytochrome *c* and Apaf-1 by combining several modeling approaches. We have applied protein-protein docking and energy minimization, evaluated the resulting models of the Apaf-1/cytochrome *c* complex, and carried out a further analysis by means of molecular dynamics simulations. We ended up with a single model structure where all the lysine residues of cytochrome *c* that are known as functionally-relevant were involved in forming salt bridges with acidic residues of Apaf-1. This model has revealed three distinctive bifurcated salt bridges, each involving a single lysine residue of cytochrome *c* and two neighboring acidic resides of Apaf-1. Salt bridge-forming amino acids of Apaf-1 showed a clear evolutionary pattern within *Metazoa*, with pairs of acidic residues of Apaf-1, involved in bifurcated salt bridges, reaching their highest numbers in the sequences of vertebrates, in which the cytochrome *c*-mediated mechanism of apoptosome formation seems to be typical.

**Conclusions:**

The reported model of an Apaf-1/cytochrome *c* complex provides insights in the nature of protein-protein interactions which are hard to observe in crystallographic or electron microscopy studies. Bifurcated salt bridges can be expected to be stronger than simple salt bridges, and their formation might promote the conformational change of Apaf-1, leading to the formation of an apoptosome. Combination of structural and sequence analyses provides hints on the evolution of the cytochrome *c*-mediated apoptosis.

**Reviewers:**

This article was reviewed by Andrei L. Osterman, Narayanaswamy Srinivasan, Igor N. Berezovsky, and Gerrit Vriend (nominated by Martijn Huynen).

**Electronic supplementary material:**

The online version of this article (doi:10.1186/s13062-015-0059-4) contains supplementary material, which is available to authorized users.

## Background

Apoptosis is a mechanism of programmed cell death that is involved in numerous processes in humans, including organism development, immune system response and aging. The intrinsic apoptotic pathway is believed to be triggered by an increased production of reactive oxygen species (ROS) in the electron-transfer chain of mitochondria, see [1–5] for reviews. One of the key subsequent events in mitochondria-mediated apoptosis is permeabilization of the inner and outer mitochondrial membranes by direct damage or by transition pore formation, followed by swelling of mitochondria [3, 6–8]. Formation of these pores, as well as rupture of the outer mitochondrial membrane, allows proteins residing in the intermembrane space to escape into the cytoplasm [9, 10].

A comparison of the intrinsic apoptotic pathways in different multicellular organisms shows that they have some common properties but also some differences [10–12]. In vertebrates, the apoptotic cascade in the cytosol is triggered by the release of cytochrome *c* from mitochondria [1, 13]. Within mitochondria, cytochrome *c* resides in the intermembrane space and transfers electrons from the ubiquinol:cytochrome *c* oxidoreductase (cytochrome *bc*_1_ complex, or respiratory Complex III) to the cytochrome *c* oxidase (respiratory Complex IV) whereby cytochrome *c* docks to acidic patches at the surface of the cytochrome *bc*_1_ complex or cytochrome *c* oxidase by using a set of positively charged lysine residues [14]. After getting into the cytoplasm, cytochrome *c* binds between the two tryptophan (W) and aspartate (D)-rich WD domains of the apoptotic protease activating factor (Apaf-1) [3, 9, 15, 16].

WD domains (also known as WD40-repeat domains) are among the top 10 most abundant domains in eukaryotic genomes and are also widespread in bacteria [17, 18]. The common function of WD domains is to serve as scaffolds for protein-protein interactions and to coordinate downstream events, such as ubiquitination or histone methylation [19]. Each WD repeat comprises a four-stranded antiparallel β-sheet secured by hydrogen bond network between the conserved residues [20]; a single WD domain is a β-propeller that can contain from 4 to 8 WD repeats as blades [21]. More generally, proteins of the β-propeller fold are widely used in nature as structural scaffolds for ligand binding, protein-protein interactions and enzymatic activity. Despite the diversity of β-propellers, their blades frequently show sequence similarity indicative of a common ancestry and are thought to be a result of independent amplification of an ancient blade-sized fragment [22, 23]. Specifically, in case of Apaf-1, cytochrome *c* binds between its 8-bladed C-terminal WD domain (hereafter WD-8 domain) and the 7-bladed WD domain (hereafter WD-7 domain) that is separated from the 8-bladed domain by a flexible loop.

Upon cytochrome *c* binding to Apaf-1, the WD-7 domain rotates to accommodate the cytochrome *c* globule between the two WD domains [24–26]. This cytochrome *c*-induced movement of WD-domains is thought to facilitate the nucleotide exchange within the *n*ucleotide *b*inding *d*omain (NBD) [25, 26]. Replacement of ADP (or dADP) nucleotide within the NBD by ATP (or dATP) molecule is associated with large rotational movement of the NBD and the neighboring helix domain 1 (HD1) [24, 25], as well as with the release of the N-terminal *c*aspase *a*ctivation and *r*ecruitment *d*omain (CARD). These events lead to the “open”, cytochrome *c*- and dATP/ATP-bound conformation of Apaf-1 proteins which then oligomerize into a heptameric platform of apoptosome [24, 27]. The CARD domains of oligomerized Apaf-1 monomers form a disc-like structure that binds the CARD domains of procaspase-9 to create asymmetric holo-apoptosome ready to activate the downstream caspases in the apoptotic cascade [25, 26, 28].

Functional studies that measured the ability of diverse cytochrome *c* variants/mutants to activate caspase-9 in the presence of Apaf-1 identified several residues of cytochrome *c* that were likely to be involved in the cytochrome *c*/Apaf-1 interaction [29–35], see also [10, 16] for comprehensive reviews. The most crucial role appeared to be played by Lys72 (hereafter, the numbering matches the mature horse [PDB:1HRC] and human [PDB:1J3S] cytochrome *c* sequences without the N-terminal methionine). Replacement of Lys72 by Arg, Trp, Gly, Leu or Ala in horse cytochrome *c* (expressed in *Escherichia coli*) led to the strongly diminished activity as compared to the wild-type [29–33]. When the metazoan cytochrome *c* was expressed in yeast cells, it got N-trimethylated in the Lys72 position and lost its ability to trigger the assembly of apoptosome [36]. Interestingly, the yeast cytochrome *c* expressed in *E. coli* was not methylated and showed certain pro-apoptotic activity, albeit well below that of the wild-type horse cytochrome *c* [29]. In addition to Lys72, mutations of residues Lys7, Lys8, Lys13, Lys25, Lys27, Lys39, Lys86, Lys87, and Lys88 were found to reduce pro-apoptotic activity of cytochrome *c* [29–35]. In some cases, the impact of mutations was shown to be additive. Specifically, Lys7Glu/Lys8Glu and Lys25Pro/Lys39His double mutants showed a ~10-fold reduction in caspase activation [29]. The only non-lysine residue mutations (of the total of 13 tested) that affected the activation of caspase were the Glu62Asn replacement in the horse cytochrome c and the mutations of the neighboring residues 63–65 [29].

The inability of the yeast cytochrome *c* with a trimethylated Lys72 and no lysine residues in positions 7 and 25 to activate vertebrate Apaf-1 [32, 36] was hardly surprising. However, the behavior of the cytochrome *c* from *Drosophila* with a set of functionally important lysine residues was more complicated. This cytochrome *c* could activate horse Apaf-1 protein and trigger the apoptosome formation [28]. Surprisingly, the same fly cytochrome *c* failed to induce caspase activation in *Drosophila* cell lysate that contained a fly homolog of Apaf-1 [9, 37, 38] capable of oligomerization into an apoptosome, which, however, contains no cytochromes *c* [39]. Apparently, while promoting the formation of an apoptosome, the lysine residues of cytochrome *c* interact with a particular set of the Apaf-1 residues, absent from the fly homolog of Apaf-1. However, as long as no sufficiently well resolved crystal structure of the cytochrome *c*/Apaf-1 complex is available, the nature of these key residues of Apaf-1 remains obscure.

**Fig. 1:**
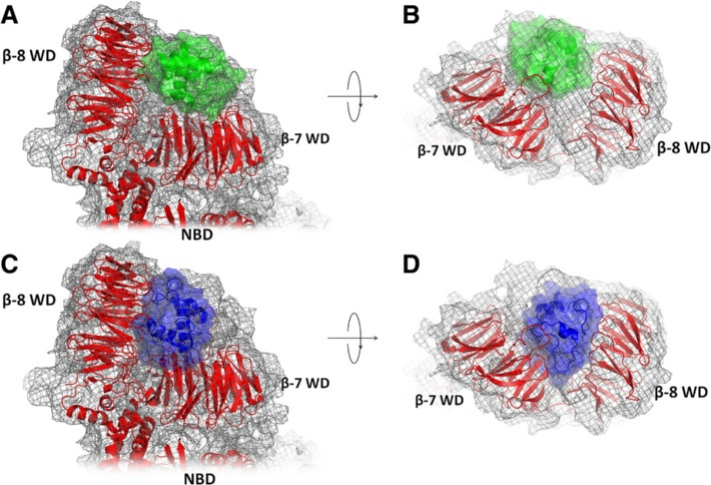
Structural models of the Apaf-1/cytochrome *c* complexes. **a, b** - the cryo-EM based model of Yuan *et al.* [PDB:3J2T] [25], top and side views; **c, d** - the Patchdock’ model (this work), top and side views. The cryo-EM map is shown as gray mesh, proteins are shown in cartoon and surface representation, Apaf-1 is red, cytochrome *c* in the cryo-EM based model [PDB:3J2T] [24] is green, the structure of cytochrome *c* in the PatchDock’ model is shown in blue

A single-particle electron density map of human apoptosome at ~9.5 Å resolution was obtained by Yuan and co-workers in 2010 [24]. Later, the same authors have improved the structure [25] by combining their single-particle electron density map [24] with the available structures of the full-length mouse Apaf-1 [PDB:3SFZ] [26], a truncated human Apaf-1 [PDB:1Z6T] [40], and the oxidized bovine cytochrome *c* [PDB:2B4Z] [41], see Fig. 1a and b. While providing powerful insight into the structure of an active apoptosome and the conformational changes in the domains of Apaf-1, this model, because of its low resolution, did not provide sufficient information either on the exact orientation of cytochrome *c* in the lobe between the two WD domains of Apaf-1 or on the residues of Apaf-1 that are involved in binding of cytochrome *c*.

In this work, we have combined several molecular modeling approaches to scrutinize the interaction between the human cytochrome *c* and the WD domains of Apaf-1. We were encouraged by recent results of Kokhan, Wraight and Tajkhorshid [42] who have studied the interaction between the yeast cytochrome *c* and the cytochrome *bc*_1_ complex using molecular dynamics (MD) simulations. Kokhan and colleagues have found that many dynamic hydrogen bonds and salt bridges, transiently showing up in their MD simulations [42], were absent from the available high-resolution crystal structures [43, 44]. Specifically, many salt bridges between the patch of lysine residues of cytochrome *c* (such as Lys79, Lys86, and Lys87) and the polar residues of the cytochrome *bc*_*1*_ complex (such as Asn169, Gln170, Asp232, Glu235, and Glu99) were shown to have a dynamic nature and were not detectable in the crystal structure [42]. The authors concluded that “the static nature of x-ray structures obscures the quantitative significance of nonbonded interactions between highly mobile residues, and that short-range electrostatic interactions are substantially involved in cyt *c* binding” [42]. These results support the earlier observations that all potential hydrogen bonds are not necessarily simultaneously present in the protein and vary depending on relevant physiological conditions [45]. The observation that even the availability of highly resolved structures does not guarantee the identification of all physiologically relevant interactions between proteins served as an additional justification for our study.

Following the approach of Kokhan and coworkers [42], we analyzed the interaction between cytochrome *c* and the WD domains of Apaf-1 by MD simulations. The surfaces of the WD domains carry a significant number of aspartate and glutamate residues, so it could be anticipated that the interaction with cytochrome *c* might be mediated by salt bridges similar to those described by Kokhan and coworkers for the interaction of cytochrome *c* with the cytochrome *bc*_1_ complex [42]. Indeed, by combining molecular modeling and MD simulations we have found a specific arrangement of cytochrome *c* between the two WD domains of Apaf-1 where cytochrome *c* was embedded in an extended network of salt bridges; these bridges involved all the lysine residues of cytochrome *c* known to be functionally important for apoptosome formation. Sequence analysis revealed a clear evolutionary pattern for the acidic residues of Apaf-1 that interacted with lysine residues of cytochrome *c* in the model structure, which may support the functional relevance of the found position of cytochrome *c* between the two WD domains of Apaf-1.

Here we scrutinized the interaction between human cytochrome *c* and Apaf-1 by combining several molecular modeling approaches with molecular dynamics simulations. The resulting model structure of the Apaf-1/cytochrome *c* complex rationalizes the literature data on functional importance of particular residues of cytochrome *c*. The identification of particular salt bridges involved in the interaction allowed us to identify the residues of Apaf-1 that might be involved in binding of cytochrome *c* and to investigate the co-evolution of the interacting residues in cytochrome *c* and Apaf-1.

## Results

### Structure analysis

The most recent model of the human apoptosome [PDB:3J2T] [25], as shown in Fig. 1a and b, contains structures of Apaf-1 in complex with cytochrome *c* that are fit into an electron density map, obtained earlier at ~9.5 Å resolution [24, 25]. The electron density map provides only the overall information about the relative location of cytochrome *c* in the cleft between the WD domains of Apaf-1. Since the Apaf-1 surface is enriched with negatively charged residues and cytochrome *c* has a plethora of lysine residues, almost any orientation of cytochrome *c* in the cleft between WD-domains of Apaf-1 would provide several salt bridges between the proteins. However, experimental data clearly indicate that this interaction is specific and requires not just a positively charged patch on the surface of cytochrome *c,* which is involved in the interaction with the cytochrome *bc*_1_ complex and cytochrome *c* oxidase, but a whole set of lysine residues located on the opposite sides of the protein globule [29–35]. This specificity of interaction implies a single functionally relevant binding mode of cytochrome *c*, which we have searched for using *in silico* approaches.

To position the cytochrome *c* molecule between the two WD domains of Apaf-1 we have started from molecular modeling. We treated the binding of cytochrome *c* to Apaf-1 as a docking problem and therefore started from using the available programs for rigid protein-protein docking and manually editing of the results obtained (see Methods). Using this approach, we obtained four predicted model structures of the Apaf-1/cytochrome *c* complex: one model by ClusPro software, one model by PatchDock software, and two models by ZDOCK software. These model structures were manually adjusted to resolve possible clashes between proteins and provide as many lysine-aspartate/glutamate pairs as possible. For the PatchDock model, the manual adjustment yielded an additional, alternative conformation (hereafter PatchDock’ structure) with cytochrome *c* that was slightly tilted respective to the original PatchDock structure. The model of the apoptosome complex obtained from the electron density map at ~9.5 Å resolution [PDB:3J2T] [25] was treated as one more model structure under investigation.

The residues 785–805 of Apaf-1 form a loop that is completely exposed to the solution and is expected to be flexible. Therefore, during manual editing, we adjusted the position of this loop in all model structures to provide salt bridge partners for the nearby lysine residues of cytochrome *c*.

All of the resulting six models placed cytochrome *c* in the lobe between two WD domains of Apaf-1 in agreement with the cryo-EM data and in each of these models the lysine residues of cytochrome *c* formed several salt bridges with Apaf-1 (Table 1).

**Table 1:** Salt bridges in putative models of Apaf-1/cytochrome *c* complex before and after energy minimization

Model	Models before energy minimization			Models after energy minimization		
	cyt *c*	Dist, Å	Apaf-1	cyt *c*	Dist, Å	Apaf-1
Cryo-EM fitting [PDB:3J2T]	GLU 90	2.80	ARG1131	GLU 90	2.68	ARG1131
	**LYS 73**	**2.58**	**ASP1023**	**LYS 73**	**2.68**	**ASP1023**
	LYS 87	3.13	ASP1106	LYS 87	3.22	ASP1064
	ARG 91	3.91	GLU1045	ARG 91	3.76	GLU1045
PatchDock model	LYS 8	3.34	ASP1106	LYS 22	2.68	ASP 903
	LYS 22	3.07	ASP 903	LYS 79	2.66	GLU 981
	LYS 79	2.68	GLU 981			
PatchDock’ model	**LYS 8**	**2.19**	**ASP1147**	**LYS 7**	**2.65**	**ASP 902**
	**LYS 72**	**3.23**	**ASP1023**	**LYS 7**	**2.77**	**ASP 903**
	LYS 79	3.07	GLU 981	**LYS 8**	**2.65**	**ASP1147**
				**LYS 25**	**2.63**	**ASP 877**
				LYS 27	3.80	GLU 925
				**LYS 39**	**2.67**	**ASP 792**
				**LYS 72**	**2.64**	**ASP1023**
				**LYS 73**	**2.63**	**GLU1045**
				LYS 79	2.63	GLU 981
ZDOCK model 1	GLU 21	2.02	LYS1004	LYS 79	2.57	ASP 902
	LYS 25	3.47	GLU 925			
	LYS 55	3.63	ASP 658			
	LYS 79	3.60	ASP 902			
ZDOCK model 2	GLU 4	3.31	HIS 840	GLU 21	2.94	LYS 802
	GLU 21	3.95	LYS 802	ASP 62	3.67	LYS1003
	GLU 61	3.28	LYS1003	LYS 5	2.63	ASP 792
	ASP 62	3.17	LYS1003	**LYS 8**	**2.64**	**GLU 798**
	LYS 5	2.47	ASP 792	**LYS 25**	**2.63**	**ASP 902**
	**LYS 25**	**3.36**	**GLU 659**	LYS 55	2.62	GLU1045
	LYS 53	3.01	ASP1106			
ClusPro model	LYS 22	3.79	GLU 981	LYS 22	2.64	GLU 981
	**LYS 25**	**2.62**	**GLU 925**	**LYS 25**	**2.62**	**GLU 925**
	HIS 33	3.48	ASP1023	HIS 33	3.48	ASP1023
	**LYS 39**	**2.69**	**ASP1147**	**LYS 39**	**2.69**	**ASP1147**
	LYS 53	2.67	ASP1147	LYS 53	2.67	ASP1147
	**LYS 73**	**2.64**	**GLU 659**	**LYS 73**	**2.97**	**GLU 659**
	LYS 79	2.60	ASP 902	LYS 79	2.60	ASP 902
	GLU 21	2.74	LYS1004	GLU 21	2.74	LYS1004

Bonds, as formed by conserved cytochrome *c* residues known to be involved in activation of the apoptosome, are **shown in bold font**

We performed energy minimization for all 6 structures and checked for salt bridges between cytochrome *c* and Apaf-1 before and after the energy minimization procedure (Table 1). After the energy minimization treatment, the models with the highest number of salt bridges involving conserved, functionally relevant lysine resides were the ClusPro server prediction and the PatchDock’ model (Table 1). Notably, the ClusPro model changed insignificantly after energy minimization, while the manually edited PatchDock’ model gained 6 new salt bridges after the energy minimization procedure (Table 1).

These two model structures were studied further by 45 ns-long free MD simulations to evaluate the stability of the obtained cytochrome *c*/Apaf-1 complexes. During the MD simulation, the domain architecture in the ClusPro model got disordered, WD domains moved apart and most of their contacts with cytochrome *c* were lost. The PatchDock’ complex remained stable (Additional file 1: Figure S1).

**Fig. 2:**
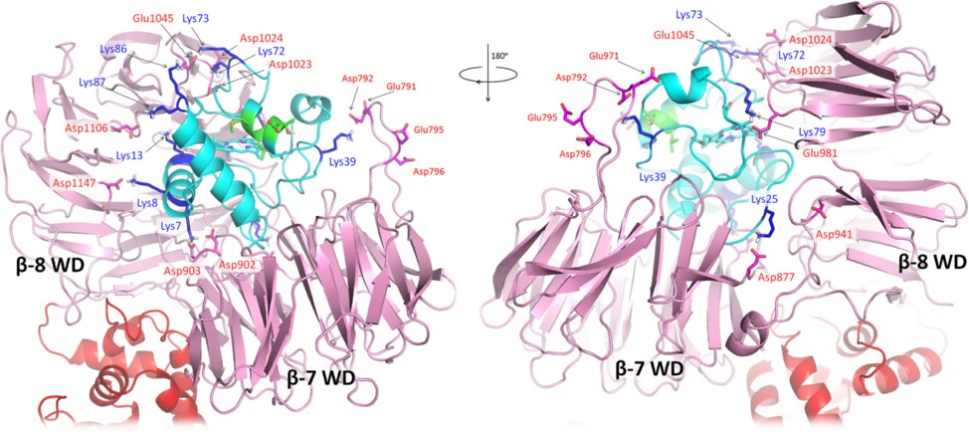
The PatchDock’ model of the Apaf-1/cytochrome *c* complex after energy minimization (see text). Contacts between cytochrome *c* and Apaf-1 are shown in blue (lysine residues) and magenta (aspartate and glutamate residies). The negatively charged patch of conserved residues 62–65 of cytochrome *c* is shown in green. The cytochrome *c* backbone and the heme are shown in cyan, the WD domains are shown in pink, and the rest of Apaf-1 monomer is colored red. Amino acid numbering is as in [PDB:3J2T]

**Fig. 3:**
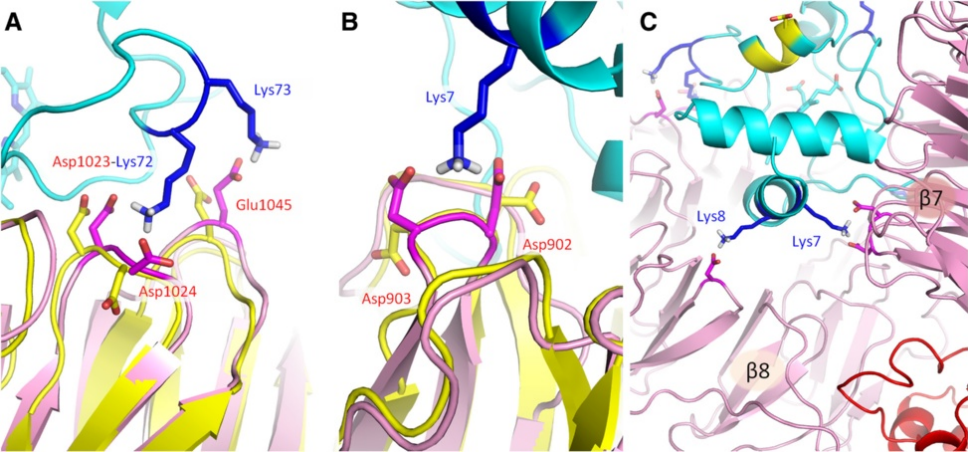
Interactions on the interface between cytochrome *c* and Apaf-1 in the PatchDock’ model (this work) and the cryo-EM based model [PDB:3J2T] [25]. Cytochrome *c* is shown in cyan, the WD domains of Apaf-1 in the PatchDock’ model are shown in pink, the WD domains in the cryo-EM based model [PDB:3J2T] [25] are shown in yellow. **a**, the network salt bridge formed by Lys72 of cytochrome *c* causes Asp1024 residue of Apaf-1 to rotate. **b**, residue Lys7 eliminates electrostatic repulsion between residues Asp902 and Asp903 of Apaf-1 by forming a bifurcated salt bridge. **c**, neighboring residues Lys7 and Lys8 create a link between two WD domains at the bottom of cytochrome *c* binding cleft. Other domains of Apaf-1 are shown in red

Thus, MD simulations revealed one model (the PatchDock’ model, Fig. 1c, d and 2) that retained the proper domain architecture and intact geometry during the MD simulation (Additional file 1: Figure S1). The same model had the largest number of stable salt bridges involving all important conserved residues of cytochrome *c* known to be involved in the interaction with Apaf-1 (Table 1, Fig. 2). These contacts involve residues at the opposite sides of cytochrome *c* globule and are evenly distributed between domains WD-7 and WD-8 of Apaf-1 (Fig. 2, Table 3). Some of these bridges are so-called complex salt bridges, involving more than two residues. In three cases, bifurcated (as defined in [46] in relation to the crystal structure of glycine [47], see also [48]), three-partite salt bridges involve a lysine amino group of cytochrome c that interacts with two neighboring acidic resides of Apaf-1. Namely, Lys72 interacts with residues Asp1023 and Asp1024 of Apaf-1 (Figs. 2 and 3a), Lys7 forms a salt bridge with the Asp902-Asp903 pair of Apaf-1 (Figs. 2 and 3b), and Lys39 forms salt bridges with the Glu791-Asp792 pair of Apaf-1 (Fig. 2). A pair of neighboring lysine residues Lys7/Lys8 provides a connection between the two WD domains, creating a relatively rigid link at the bottom of the cytochrome *c* binding pocket (Figs. 2 and 3c).

**Fig. 4:**
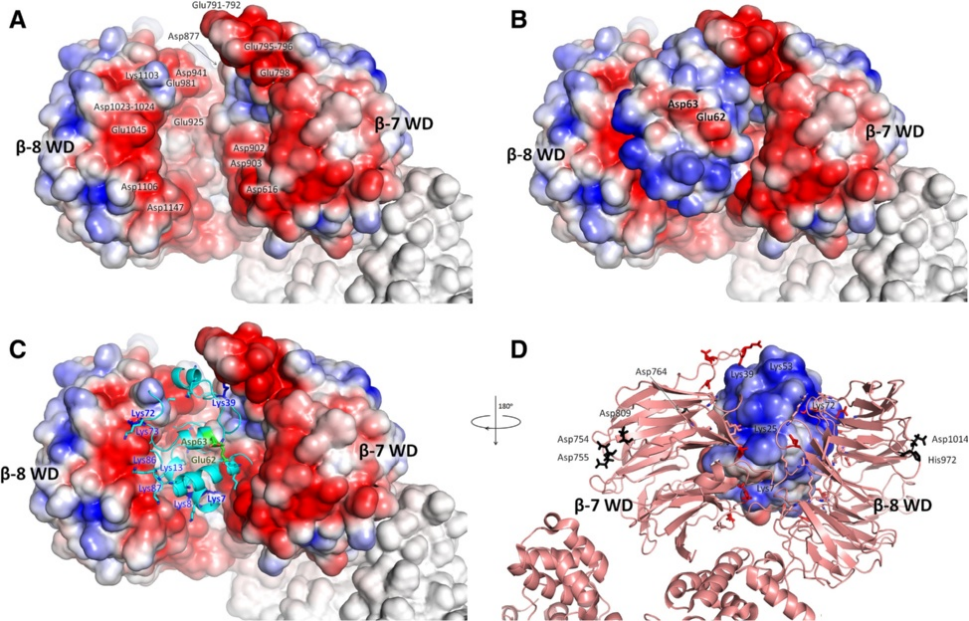
Electrostatic properties of the interacting surfaces of Apaf-1 and cytochrome *c* as calculated with the APBS (Adaptive Poisson-Boltzmann Solver [77]) and PDB2PQR [75, 76] software packages. The linear color scale was set from −3 (red) to 3 (blue) kcal/mol. **a**, WD domains of Apaf-1 are shown in a surface representation colored according to electric charge (red, negative; blue, positive), other domains of Apaf-1 are not colored, cytochrome c is not shown to reveal the negative charge of the binding interface; **b**, Surfaces of cytochrome *c* and WD domains of Apaf-1 are shown simultaneously, the negatively charged spot (colored red) on the cytochrome *c* surface is facing the outside; **c**, cytochrome *c* is shown in a cartoon representation with lysine residues shown as sticks (conservative residues shown in blue) and conserved residues 62–65 matching the negatively charged spot shown in green; **d**, the cytochrome c/Apaf-1 complex is shown in a “back view”, rotated by 180° as compared to panels **a**–**c**. Apaf-1 is shown in a cartoon representation, the acidic surface residues of WD domains potentially accessible to cytochrome *c* are shown as red sticks, the conservative acidic residues that are remote from the cytochrome c binding interface of the WD domains are shown as black sticks.

We also calculated the electrostatic properties of the human cytochrome *c* and Apaf-1 (see Fig. 4). While the surface of the cleft between the two WD domains of Apaf-1 is negatively charged, the surface of cytochrome *c* is mostly positively charged but has a distinct negatively charged patch that corresponds to Asp62 and neighboring residues. The Glu62Asn replacement at this position and mutations of the neighboring residues 63–65 are the only non-lysine mutations that are known to affect the activation of Apaf-1 [29] (the horse cytochrome *c* sequence, used in these experiments, contains a glutamate residue in the 62nd position, while the human cytochrome *c* has an aspartate). In the PatchDock’ model, this negatively charged area on cytochrome *c* surface is facing outside from the WD domains cleft (Fig. 4).

The PatchDock’ structure showed a good fit to the experimental electron density map with correlation coefficient of 0.9463 as compared to 0.9558 for the model structure that had been obtained earlier from cryo-EM data by Yuan *et al.* [PDB:3J2T] [24, 25], see Fig. 1. However, the cytochrome *c* position appears to be different in the two models. In the PatchDock’ structure, the cytochrome *c* globule sits deeper in the lobe between the two WD domains (Fig. 1c and d), while in cryo-EM-based structure of Yuan *et al.* [PDB:3J2T] [25] cytochrome *c* is not reaching the bottom of the lobe between two WD domains, which leaves some unoccupied electron density (Fig. 1a and b). In contrast, the deeper position of cytochrome *c* in the PatchDock’ model results in an unoccupied density in the cryo-EM map close to the surface of the WD domains (Fig. 1c and d).

For all model structures that were obtained after energy minimization, as well as for the cryo-EM-based structure by Yuan *et al.* [25], we have estimated the change in the solvation energy upon the binding of cytochrome *c* to Apaf-1 (ΔG^s^), see Table 2. All model structures that were obtained via docking programs and the subsequent editing procedures showed negative values of ΔG^s^ (Table 2). Most beneficial interaction interfaces were provided by the model structures ZDOCK1 and PatchDock’. The cryo-EM-based structure of Yuan and co-workers [25] was characterized by a positive value of ΔG^s^ (Table 2). In addition, by using the same approach, we calculated, for comparison, the values of ΔG^s^ for the available high-resolution structures of the complexes of cytochrome *c* with the cytochrome *bc*_1_ complex (see Table 2).

**Table 2:** Properties of structural models of the cytochrome c/Apaf-1 complex as compared to the crystallographic data on the interactions of cytochrome *c* with the cytochrome *bc*_1_ complex

Structure	Solvation energy of the complex formation, kcal/mol	Interface area, as % of the total solvent-accessible surface of cytochrome *c*
**Apoptosome complex**		
3j2t	1.4	16.0 %
cluspro	−4.8	23.2 %
patchdock	−5.9	26.2 %
patchdock’	−6.2	32.1 %
zdock1	−14.3	28.8 %
zdock2	−4.8	30.0 %
**Cytochrome bc1 complex**		
1kyo crystal	−1.6	9.9 %
3cx5 crystal	−1.2	9.9 %

For all structures listed in Table 2, we have also calculated the fraction of cytochrome *c* surface involved in the interactions with the domains of Apaf-1 and with the surface of the cytochrome *bc*_1_ complex, respectively (see Table 2).

**Fig. 5:**
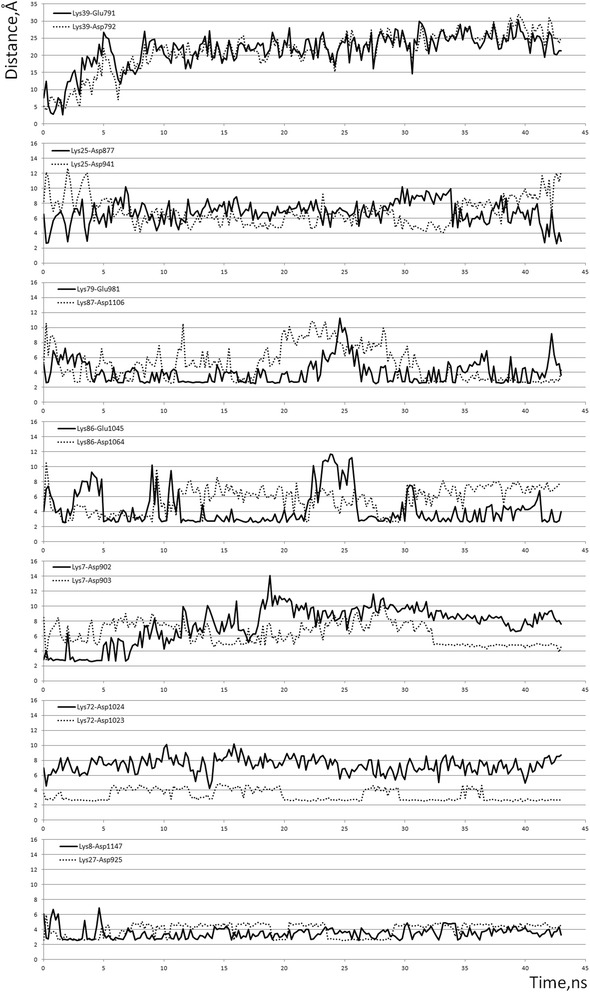
Distances between the charged groups involved in ionic bonds between cytochrome *c* and Apaf-1, as measured during the free MD simulation. Distances were measured between the nitrogen atoms of the amino groups of lysine side chains and the closest oxygen atoms of the side chains of aspartate and glutamate residues of Apaf-1

**Fig. 6:**
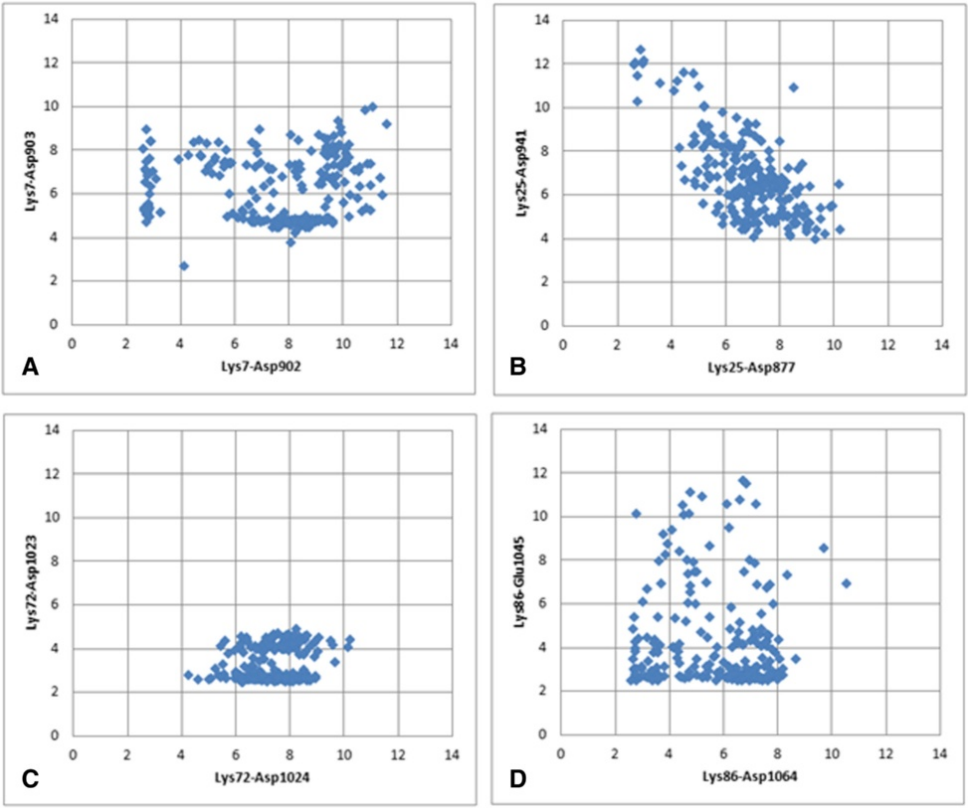
Locations of a lysine amino group in relation to carboxyl groups in bifurcated salt bridges. Distances (in Å) were measured between nitrogen atoms of side chain amino groups of cytochrome *c* lysine residues and the closest of side chain oxygen atoms of aspartate or glutamate residues of Apaf-1

**Fig. 7:**
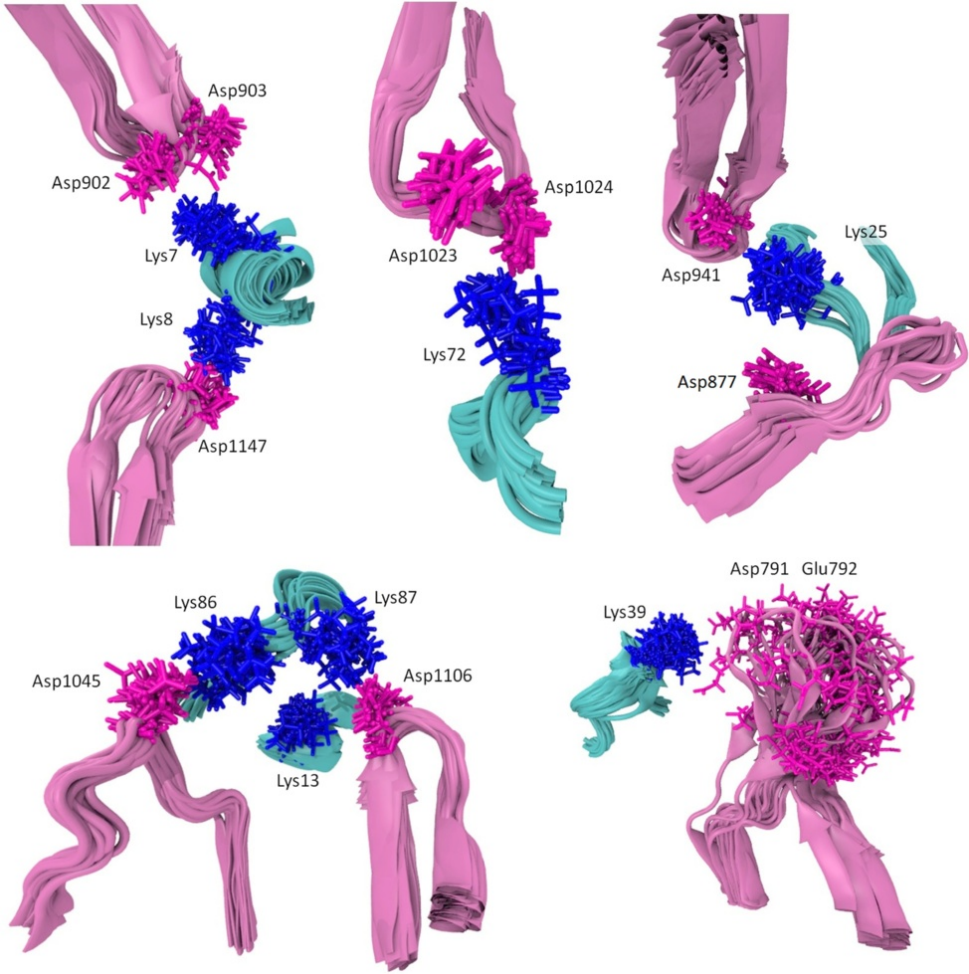
Mobility of complex salt bridges between cytochrome *c* and Apaf-1 in the course of MD simulations. Conformations of particular complex salt bridges observed in MD simulation were superimposed individually for each group of contacts. Protein backbone fragments are shown in cartoon representations: cytochrome *c* in cyan, Apaf-1 in magenta. Interacting residues are shown in stick representation: lysine residues in blue, aspartate and glutamate residues in red

Molecular dynamics simulations provided an ensemble of conformations which allowed further evaluation of stability of the protein contacts and of their impact on the structure of the PatchDock’ complex (Figs. 5, 6 and 7). Salt bridges, including the H-bonded interactions, are generally considered within a 4 Å cutoff [42, 49–54]. However, interactions over 4 Å should not be disregarded. Lee et al. described the distance dependence of electrostatic interactions between charged amino acids by a coulombic function that captured the experimental and theoretical data [55]. Pairwise coulombic interactions among surface charges were significantly weakened at distances of interaction greater than 5 Å. Under conditions of “physiological” ionic strength (0.1 M), the energy of interaction between charges separated by 10 Å was approximately 0.1 kcal/mol, but increased up to 0.5–1.0 kcal/mol already when the charges were separated by 5 Å [55]. At the binding interface, we observed both short and long-range interactions between charged residues (Table 3). Each of the salt bridges in Table 3 became shorter than the classical 4 Å cutoff [42, 43, 49–53], at least transiently, during the MD simulation (Fig. 5).

**Table 3:** Salt bridges between Apaf-1 and cytochrome *c* in the PatchDock’ model

Amino acid pair	Distance, Å		Contact time during simulation			
	after energy minimization	during MD simulation	<3 Å	<4 Å	<5 Å	<6 Å
Lys7-Asp902	2.65	7.55 ± 2.44	12 %	13 %	17 %	24 %
Lys7-Asp903	2.77	6.34 ± 1.48	0 %	1 %	32 %	47 %
Lys8-Asp1147	2.65	3.51 ± 0.75	29 %	78 %	97 %	98 %
Lys25-Asp877	2.63	6.80 ± 1.48	3 %	4 %	11 %	25 %
Lys25-Asp941	11.37	6.87 ± 1.85	0 %	0 %	14 %	34 %
Lys27-Glu925	3.8	3.98 ± 0.89	28 %	34 %	99 %	100 %
Lys39-Glu791	2.67	21.52 ± 5.12	1 %	1 %	2 %	2 %
Lys39-Asp792	12.44	21.25 ± 5.99	0 %	0 %	2 %	3 %
Lys72-Asp1024	4.57	7.37 ± 1.02	0 %	0 %	1 %	8 %
Lys72-Asp1023	2.64	3.32 ± 0.77	57 %	69 %	100 %	100 %
Lys79-Glu981	2.63	4.06 ± 1.57	40 %	53 %	78 %	88 %
Lys86-Glu1045	7.01	4.37 ± 2.26	38 %	60 %	77 %	81 %
Lys86-Asp1064	8.95	5.67 ± 1.73	9 %	23 %	37 %	47 %
Lys87-Asp1106	9.56	5.11 ± 2.29	27 %	41 %	56 %	69 %

Distances between amino group nitrogens and the nearest of two carboxyl group oxygens are given for the structure after energy minimization (static parameter) and in the course of the MD simulation (dynamic parameter)

All the interactions listed in Table 3 stayed throughout the 45-ns free MD simulation, with the exception of Lys39 contacts with residues Glu791 and Asp792, which got broken (Fig. 5, the top trace, and Fig. 7). The residues Glu791–Asp792 of Apaf-1 are part of a loop that comprises residues 785–805; this highly mobile loop floated as a whole away from cytochrome *c* surface during the MD simulation (see also Additional file 1: Figure S1). Generally, the dynamic behavior of said bonds was mainly due to the side chain fluctuations and was not notably influenced by protein backbone mobility, with the exception of contacts formed by Lys39 (Fig. 7). However, neither of the observed contacts was long-living. Instead, each particular contact was lost and then regained at picoseconds. The only exceptions were the salt bridges between residues Lys25 and Asp941 as well as Lys8 and Asp1147, which could be maintained for up to 10 ns (Fig. 5).

Figure 2 reveals multiple bifurcated salt bridges that involve a single lysine residue of cytochrome *c* as a proton donor and carboxyl groups of two aspartate or glutamate residues of Apaf-1 as proton acceptors. In addition to the three aforementioned bridges where the lysine residues of cytochrome *c* interact with pairs of neighboring acidic residues of Apaf-1, there are also interactions of Lys25 with Asp877 and Asp941, and Lys86 with Asp1064 and Glu1045 (see Table 3). In some of these bifurcated bonds the hydrogen bonds are not equivalent, so that the strong (“major”) and weak (“minor”) components can be identified. To describe the components of bifurcated salt bridges, we have plotted the distances from each proton donor group to the two available acceptors against each other (Fig. 6).

The interaction of Lys7 with Asp902 and Asp903 (Fig. 6a) shows two distinct states, characterized by a lysine residue shifted to either one or the other aspartate residue, respectively. However, the population of these states is low (13 % for the conformations with Lys7 shifted to Asp902, and 26 % for the conformations with Lys7 shifted to Asp903); in all the other conformations the amino group of Lys7 is “scattered” between the two carboxyl groups. In contrast, the interactions of Lys25 residue with Asp877 and Asp941 (Fig. 6b) are not characterized by distinct states. The interactions of Lys72 with Asp1023 and Asp1024 (Fig. 6c) are shifted in favor of forming a salt bridge between Lys72 and Asp1023, which can be considered a major state in this case. The interactions of Lys86 with Asp1064 and Glu1045 are biased in favor of a salt bridge between Lys86 and Glu1045 (Fig. 6d).

**Fig. 8:**
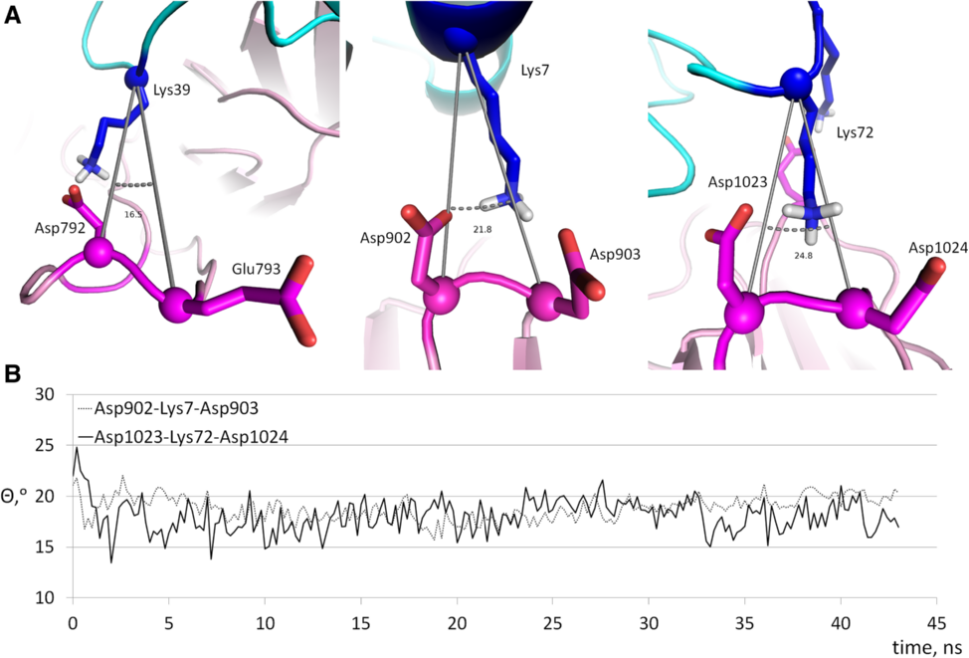
Geometry of bifurcated salt bridges. **a**, Values of the angle between Cα atoms for complex salt bridges in the PatchDock’ model structure after energy minimization. **b**, Values of the angle between Cα atoms for the same structure during the MD simulation. Values for the Asp792-Lys39-Glu793 salt bridge are not shown due to the high mobility of the respective loop of Apaf-1 (residues 785–805)

An important geometrical feature of bifurcated, complex salt bridges is the angle Θ between the Cα atoms of interacting amino acids [53]. We measured the Θ angles in the PatchDock’ model structure after energy minimization and during the MD simulations to establish whether the bifurcated salt bridges in the model were cooperative or not. The small values of the Θ angles (Fig. 8) indicate high cooperativity of the salt bridges, see also the Discussion section.

### Sequence analysis

To substantiate the deduced structure of the complex between cytochrome *c* and Apaf-1, we performed a comparative analysis of the cytochrome *c* and Apaf-1 sequences in different organisms.

**Fig. 9:**
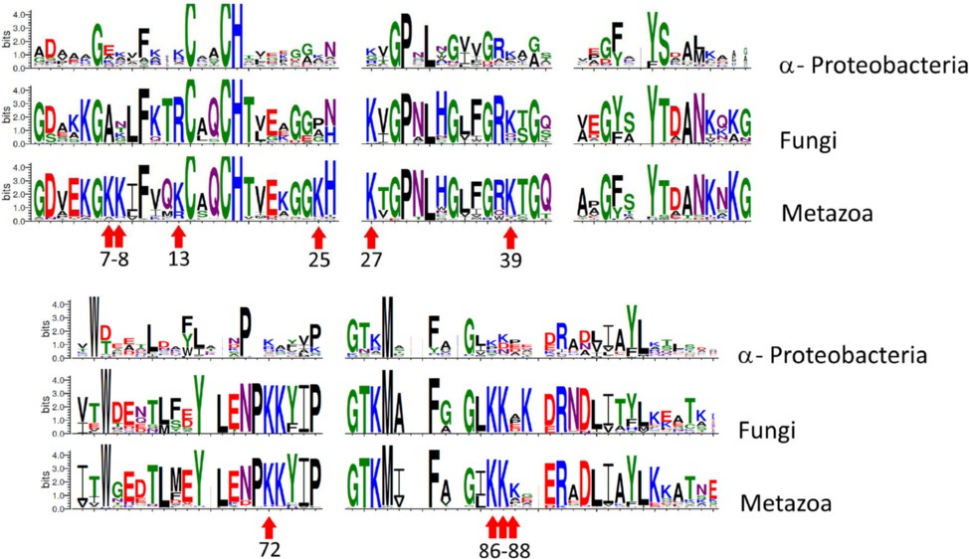
Conservation of the positively charged residues in the cytochrome *c* sequences. Sequence logos were generated with WebLogo [89] from multiple alignments of bacterial and eukaryotic cytochrome *c* sequences from fully sequenced genomes. The numeration of residues corresponds to the mature human cytochrome *c*. Each position in the logo corresponds to a position in the alignment while the size of letters in the position represents the relative frequency of corresponding amino acid in this position. Red arrows indicate residues experimentally proven to be involved in interaction with Apaf-1

Upon PSI-BLAST search of cytochrome *c* sequences in the RefSeq database, 168 proteobacterial, 56 fungal, and 209 metazoan sequences were retrieved after the third iteration. Multiple alignments of these three groups were used to obtain the logo diagrams (Fig. 9). As already noted, an interesting feature of the cytochrome *c* sequences is the presence of a set of positively charged lysine residues which interact with the negatively charged “docking” patches at the surface of its functional partners [14]. We have checked how this pivotal set has evolved. As shown in Fig. 9 by arrows, the number of lysine residues has increased in the course of evolution from proteobacteria to *Metazoa*. Apparently, the higher number of lysine residues facilitated the binding of cytochrome *c* to its functional targets.

**Fig. 10:**
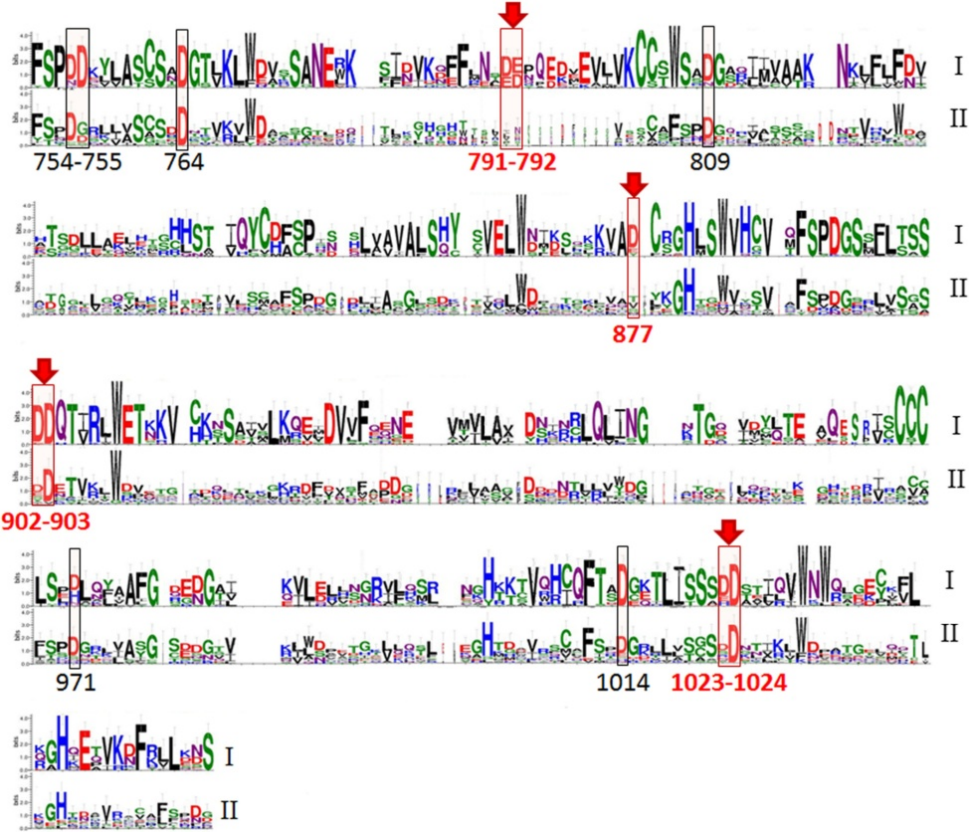
Conservation of negatively charged residues in the sequences of Apaf-1 homologs. The numeration of residues corresponds to the human Apaf-1. Sequence logos were generated with WebLogo [89] from multiple alignments of 22 sequences from group I, which included Chordates (Vertebrates and Cephalochordates), and 15 sequences from group II (Hemichordates, Echinoderms, Platyhelminthes, Cnidaria, Arthropods, and Placozoa). Each position in the logo corresponds to a position in the alignment while the size of letters in the position represents the relative frequency of corresponding amino acid in this position

We also performed a comparative sequence analysis of the Apaf-1 proteins (Fig. 10 and Additional file 1: Figure S2). Using our model of the cytochrome *c*/Apaf-1 complex, we have traced the evolution of acidic residues of Apaf-1 that were involved in formation of the salt bridges in the PatchDoc’ structure and checked for correlation with the evolution of the functionally important cytochrome *c* lysine residues. The Apaf-1 residues involved in cytochrome *c* binding in the PatchDock’ model are conserved among the vertebrates, in agreement with the common apoptosome assembly pathway and conserved cytochrome *c* residues (red arrows in Fig. 10). The Apaf-1 sequences of planarian flatworm *Schmidtea mediterranea* and sea urchin *Strongylocentrotus purpuratus* (phylum *Echinodermata*), for which the cytochrome-dependent apoptosome formation has been shown [12], contain some of these acidic residues, but not all of them (see in the Additional file 1: Figure S2). Noteworthy, the Asp1023-Asp1024 pair that forms a salt bridge with Lys72 in the PatchDock’ structure is one of the most conserved, with at least one aspartate residue being present generally in all *Metazoa*, which mirrors the conservation of Lys72 residue through all *Metazoa* as well (Fig. 9). A peculiar replacement of one aspartate in this pair to histidine is observed in *Aves* (birds), although apoptotic pathways have only been well studied in chicken cells, and the chicken Apaf-1 has aspartates or glutamates in all positions proposed to be important for apoptosome assembly. For the 791–792 and 902–903 pairs of acidic residues there is a clear evolutionary trend of their prevalence within chordates. A comparison of Figs. 9 and 10 shows that while proteins with the Apaf-1 domain architecture are already seen in *Nematostella vectensis* and *Trichoplax adhaerens*, the set of potent ligands of cytochrome *c* described in this work has fully evolved at the level of *Сhordates*, probably after their branching from *Echinoderms and Hemichordates*.

## Discussion

In this work, we present a model of the Apaf-1/cytochrome *c* complex which could serve as a basis for detailed investigation of specific interactions that underlie the apoptosome assembly. For the lysine residues that are known to be crucial for the ability of cytochrome *c* to induce apoptosis, we have identified acidic counterparts in Apaf-1. In three cases, acidic “duplets” (pairs of adjacent aspartate and/or glutamate residues) were involved in complex salt bridges with lysine residues of cytochrome *c*.

We estimated the changes in the solvation energy due to the interface formation (ΔG^s^), as well as fractions of the cytochrome *c* surface involved in the interaction with Apaf-1 for all the model structures including the one that had been obtained earlier from cryo-EM data by Yuan and co-workers [25], see Table 2. For all our model structures the calculated values of solvation energies ΔG^s^ were distinctly negative, unlike the cryo-EM-based structure of Yuan and co-workers [25] for which the ΔG^s^ value was positive (Table 2). This positive value correlated with the smallest fraction of cytochrome *c* surface involved in the interactions with the domains of Apaf-1 in this structure as compared with the model structures that were obtained by using docking programs (see Table 2). It is noteworthy that the cryo-EM-based model structure of Yuan and co-workers was obtained by maximizing the correlation with electron density as experimentally measured in [24], while our model structures were obtained by docking methods that generally search for maximal energy gains and the largest interaction interfaces for the docking partners. The PatchDock’ model structure showed the largest interaction surface.

The smaller, albeit negative values of ΔG^s^, as calculated for the high-resolution complexes of cytochrome c with the cytochrome *bc*_1_ complexes (Table 2) can be explained by smaller interactions surfaces: while in the cytochrome *c/*Apaf-1 complex both sides of cytochrome *c* interact with the domains of Apaf-1, only one side of cytochrome *c* interacts with the cytochrome *bc*_1_ complex.

The role of the conserved negatively charged patch of residues 62–65 in the PatchDock’ structure might be in providing orientation of cytochrome *c* in its binding cleft between the two negatively-charged surfaces of the Apaf-1 domains. Noteworthy, this region faces away from the contact interface, as it also does in the complexes of cytochrome *c* with the cytochrome *bc*_*1*_ complex [43].

All of the initial six models placed cytochrome *c* in the lobe between two WD domains of Apaf-1, in agreement with the cryo-EM data, and in each of these models lysine residues of cytochrome *c* formed salt bridges with Apaf-1. However, only some of these models invoked the functionally important lysine residues and only the PatchDock’ model included a salt bridge formed by Lys72 from the very beginning (Table 1).

Specifically, the position of the functionally important Lys72 residue in the PatchDock’ structure indicates the possibility of a complex salt bridge formation with the Apaf-1 residues Asp1024 and Asp1023 (Fig. 3a), although in the latter case the 4.6 Å distance between the charged moieties after energy minimization is larger than usually expected for salt bridges (see the discussion of the cut-off distances below). In contrast, in the model of Yuan and colleagues [PDB:3J2T] [25], it is the neighboring residue Lys73 that is forming the salt bridge with Asp1023, while Lys72 of cytochrome *c* and Asp1024 of Apaf-1 are facing away from interaction interface. It is tempting to speculate that binding of Lys72 might play a guiding role in docking of cytochrome *c* to Apaf-1.

Interactions involving more than two charged residues are commonly referred to as “complex” or “networked” salt bridges. Complex salt bridges have been investigated for their role in stabilizing protein structure and protein-protein interactions [52, 56–60]. While playing an important role in connecting elements of the secondary structure and securing inter-domain interactions in proteins, complex salt bridges are commonly formed by partners that are separated by 3–4 uninvolved residues in the protein chain. Repetitive cases within the same protein domain with neighboring residues of the same charge being involved in bifurcated interactions, three of which are predicted in the PatchDock’ structure, to the best knowledge of the authors, have not been reported until now. This is not surprising, since the repulsion between two negatively charged residues could hardly contribute to the protein stability [61]. Still, in the case of Apaf-1, there is a clear pattern of emergence and evolutionary fixation of several Asp-Asp motifs (Fig. 10) that, as the modeling suggests, might be involved in binding the lysine residues of cytochrome *c*.

The geometry of the interactions between acidic and basic residues is similar in simple and complex salt bridges. Adding a residue to a simple interaction represents only a minor change in the geometry but yields a more complex interaction, a phenomenon that may explain the cooperative effect of salt bridges in proteins. Energetic properties of complex salt bridges vary depending on the protein environment around the salt bridges and the geometry of interacting residues. Detailed analyses of the net energetics of complex salt bridge formation using double- and triple-mutants gave conflicting results. In two cases, complex salt bridge formation appeared to be cooperative, i.e., the net strength of the complex salt bridge was more than the sum of the energies of individual pairs [62, 63]. In one case, formation of a complex salt bridge was reported to be anti-cooperative [64].

Statistical analysis of complex salt bridge geometries performed on a representative set of structures from the PDB revealed that over 87 % of all complex salt bridges formed by a basic (Arg or Lys) residue and two acidic partners have a geometry such that the angle formed by their Cα atoms, Θ, is < 90° [53]. Same preferred geometry was observed in the two aforementioned instances when the energetics of complex salt bridge formation was cooperative [62, 63], while in the reported anti-cooperative complex salt bridge [64] the value of Θ was close to 160°. The anti-cooperativity of complex salt bridges with Θ = 150° was also established by measuring the stability of model proteins [53].

It is noteworthy that complex salt bridges can be also found at the interfaces of cytochrome *c* with other proteins; due to dynamic nature of such interactions they are not always reflected in crystallographic structures. Crystal structures are available for cytochrome *c* bound to the cytochrome *bc*_1_ complex [43, 44], the cytochrome *c* peroxidase [65], the photosynthetic reaction center [66], along with a theoretical model of the complex with cytochrome *c* oxidase [67]. Most of interactions described for cytochrome *c* lysine residues can be classified as long-distance electrostatic interactions with distances between charged groups in the 4 to 9 Å range [43, 44, 65–67]. Still, some of these interactions involve pairs of negatively charged residues, and in few cases – even pairs of neighboring residues [44].

The geometry of bifurcated salt bridges in the PatchDock” model of the Apaf-1/cytochrome *c* complex shows surprising resemblances to the known cytochrome *c* interactions with other partners*.* For example, on the interface between cytochrome *c* (chain W in [PDB:3CXH]) and cytochrome *c*_1_ of the yeast cytochrome *bc*_1_ complex (chain O in [PDB:3CXH]) the bifurcated salt bridge between Lys96 (Lys87 in human) of cytochrome *c* and the duplet of aspartate residues of cytochrome *c*_1_ (Asp231 and Asp232) shows Θ = 22.8°. This value indicates cooperativity between the bonds involved in these interactions. The bifurcated salt bridges in the PatchDock’ cytochrome *c*/Apaf-1 complex, described above, show pretty small values for the Θ angle, around 15–20° (Fig. 8). According to Gvritishvili *et al*. [53], such small angles would indicate high cooperativity for these bonds. However, an important destabilizing factor in this interaction might be the conformational tension in the protein backbone. The bifurcated salt bridges reported here include acidic residues located next to each other on relatively loose loops between the β-strands of WD domains, so the energetic gain upon insertion of a positive charge between two negatively charges moieties can be accompanied by a loss in protein backbone mobility. In addition, with the introduction of a positively charged lysine residue, the carboxyl groups of two Asp residues are being forced to come closer together (Fig. 3a and b), which might create tension in the protein backbone structure and trigger certain conformational changes in the Apaf-1 protein.

Within Apaf-1, the signal about the binding of cytochrome *c* to the WD domains should be mechanistically transmitted towards the nucleotide-binding domain. Formation of bifurcated salt bridges may be involved in this signaling, since such interactions: (i) are specific to the apoptotic pathway; (ii) should cause conformational changes in those loops that carry the neighboring pairs of acidic residues (Fig. 3a and b); and (iii) might be energetically favorable to an extent sufficient to initiate a conformational rearrangement of the whole Apaf-1 structure enabling transmission of a signal to the partner from the other side of the WD domain.

We would like to emphasize that our structure, as shown in Figs. 1c, d, 2, and 4 is just a theoretical prediction; the ultimate structural solution of the Apaf-1/cytochrome *c* complex would come, hopefully, in the near future, along with a well-resolved crystal and/or cryo-EM structure of the complex. Although we hope that this structure would match our prediction, there is obviously no guarantee. Taking into account the large number of lysine residues that are spread all over the surface of cytochrome *c*, one could not exclude some alternative arrangement of cytochrome *c* between the two WD domains, which also would satisfy the existing functional constrains. It also seems plausible that binding of cytochrome *c* between the two WD domains, as well as its release from a mature holo-apoptosome, might both be multistep processes, so that the structure in Fig. 1 might correspond to only one of the structural intermediates. Our goal was, however, to identify the residues of Apaf-1 that are involved in binding of cytochrome *c*. Accordingly, we believe that the acidic “duplets”, which are particularly abundant in the Apaf-1 sequences of vertebrates, would withstand the scrutiny of further experimental studies as the key players in promoting the apoptosome formation.

Replacement of key lysine residues of cytochrome *c* has been shown to decrease its ability to cause caspase activation [29–35]. Accordingly, the appearance of these lysine residues at the surface of cytochrome *c* in the course of evolution (Fig. 9) should have increased the ability of cytochrome *c* to promote apoptosis - provided that new acidic counterparts for these lysine residues emerged concurrently on the interacting surfaces of the WD domains, which seems to be the case, *cf* Fig. 9 with Fig. 10 and Additional file 1: Figure S2. Bifurcated salt bridges, which should be stronger than the simple ones, could further contribute to the ability of cytochrome *c* to promote apoptosome formation.

This scenario, as well as our model, lead to an experimentally testable prediction that replacement of the acidic residues of Apaf-1, identified in this work, would decrease the ability of cytochrome *c* to promote apoptosis. Such experimental validation might be useful also for other WD domains (tryptophane and *aspartate*-rich) as salt bridges formed by these acidic residues might account for the ability of these domains to mediate protein-protein interactions also in other cell systems.

While the number of acidic residues of Apaf-1 in the regions facing cytochrome *c* is increased in vertebrates as compared to other taxa, there are also conserved aspartate residues on the sides of WD domains that are opposite to the cytochrome *c*-interacting sides (black boxes in Fig. 10). As these residues cannot be involved in the binding of cytochrome *c*, their conservation, perhaps, indicates their involvement in the interaction of Apaf-1 with some other partner (s). Several proteins, besides cytochrome *c*, can bind to Apaf-1 and affect its activation, see [68] for a review. For example, specific binding to the WD domains of Apaf-1 was demonstrated for the anti-apoptotic Bcl-2 family member Boo [69]. Particularly interesting are the positions 754 and 755 of Apaf-1 (Figs. 4 and 10) where a clear evolutionary trend of emergence of an aspartate duplet can be seen. These aspartate residues are very likely to bind one of the Apaf-1-modulating proteins.

WD-40 repeat-containing proteins are abundant among the conserved clusters of orthologous groups of eukaryotic proteins [70]. These proteins are subunits of major, eukaryote-specific protein complexes, such as the rRNA processosome [71], and the presence of numerous paralogs indicates that architecture of these complexes, with the unique functions of individual subunits, almost entirely evolved at a very early stage of eukaryotic evolution via multiple duplications of genes for superstructure-forming proteins [72]. Thus, all numerous paralogous proteins containing WD-40 repeats are expected to function as structural components of multisubunit complexes [72], with WD domains mediating interactions between protein domains [25, 73, 74], the function that we have addressed here on the example of Apaf-1. It is tempting to speculate that WD domains, generally, mediate interactions between proteins by changing their conformation in response to various impacts that affect the acidic residues of the loops that connect the rigid β-blades.

## Conclusions

Here we have combined structural and phylogenetic analyses with MD simulations to clarify the interactions of cytochrome *c* with Apaf-1. The obtained model of the cytochrome *c* / Apaf-1 complex fits into the experimental electron density map of the apoptosome and provides acidic salt bridge partners for all the lysine residues that are known to be crucial for the ability of cytochrome *c* to induce apoptosis. It appears that in the course of evolution, binding of cytochrome *c* to Apaf-1 has improved not only due to an increase in the number of lysine residues of cytochrome *c* that are involved in binding to Apaf-1, but also through the emergence of aspartate pairs in Apaf-1, which enabled the formation of complex, bifurcated salt bridges with those lysine residues. Uncovering the details of the involvement of the bifurcated salt bridges in triggering the apoptosome formation would require studying the interactions of WD domains with other domains of Apaf-1; such investigations might shed light on the overall energy balance of the apoptosome assembly.

## Methods

### Structures used

We used coordinates of the full-length human Apaf-1 protein in cytochrome *c*-bound state [PDB:3J2T] [25] and the NMR solution structure of reduced human cytochrome *c* [PDB:1J3S] (Jeng WY, Shiu JH, Tsai YH, Chuang WJ. 2009. Solution structure of reduced recombinant human cytochrome *c*, unpublished).

### Electrostatic calculations

We used the APBS (Adaptive Poisson-Boltzmann Solver) and PDB2PQR software packages designed for analysis of the solvation properties of small and macro-molecules such as proteins, nucleic acids, and other complex systems. We used PDB2PQR [75, 76] to prepare the setup (structures and parameters) for calculations, and APBS [77] for modeling properties of protein surfaces by solving the Poisson-Boltzmann equation. We used the versions implemented as web servers hosted by the National Biomedical Computation Resource (http://nbcr-222.ucsd.edu/pdb2pqr_2.0.0/). Protonation states of residues were assigned with the PROPKA software [78], separately for the Apaf-1 and cytochrome *c* structures.

### Modeling of the cytochrome *c* binding to Apaf-1

To predict the orientation of cytochrome *c* in its binding cleft we used several rigid protein-protein docking software packages that are based on different approaches, namely PatchDock [79], ZDOCK [80], and ClusPro [81], and combined them with manual editing and evaluation of the obtained models.

The *PatchDock* algorithm is inspired by object recognition and an image segmentation technique used in computer vision and applies geometric hashing and pose-clustering matching to match convex and concave patches of interacting surfaces [79]. The web server is located at http://bioinfo3d.cs.tau.ac.il/PatchDock/.

*ZDOCK* is a fast Fourier transform (FFT)-based protein docking program which searches all possible binding modes in the translational and rotational space between the two proteins and evaluates each pose using an energy-based scoring function [80]. The web server is at http://zdock.umassmed.edu/.

*ClusPro* also uses the FFT-based rigid docking with an addition of low energy results clustering under the assumption that a native binding site will have a wide free-energy attractor with the largest number of results [81]. The web server is at http://cluspro.bu.edu/.

Additionally, the orientation of cytochrome *c* in the cryo-EM fitted structure of apoptosome [PDB: 3J2T] [25] was also treated as a model under investigation.

The software that we used for calculating the protein-protein docking operates with rigid bodies (ZDOCK and PatchDock servers) or incorporates only side-chain flexibility (ClusPro). Thus, we used manual editing, energy minimization procedure, and, at the final stage, free molecular dynamics simulations to refine the model structures and examine the flexible interacting interfaces.

*Structure editing and evaluation* were done manually using PyMOL [82]. During the analysis of the obtained structural models we were mainly considering the number of salt bridges and hydrogen bonds between the interacting proteins. At each stage of modeling we used the PISA service at the European Bioinformatics Institute (http://www.ebi.ac.uk/pdbe/pisa/) [83] to list salt bridges and hydrogen bonds between the proteins in the complex (Table 1). PISA was also used for estimating the change of the solvation energy of the cytochrome *c* structure due to the interface formation (ΔG^s^) (Table 2), as well as the fraction of cytochrome *c* surface involved in the interactions with Apaf-1 and the cytochrome *bc*_1_ complex, respectively (Table 2).

We have used the UCSF Chimera package [84] to fit the model structures into the experimental cryo-EM data [24] and to calculate the correlation coefficients.

### Molecular dynamics (MD) simulations

For the MD simulations we used the Gromacs v.4.5.5 software with MPI implementation at the supercomputer SKIF “Chebyshev” (the Computational Center of the Lomonosov Moscow State University). The protein molecules were modeled with the CHARMM36 force field. The system for simulation consisted of an Apaf-1/cytochrome *c* complex placed in the simulation box that was big enough to provide at least 12 Å distance from protein atoms to periodic cell walls. Each model was placed in a water box with addition of Na^+^ and Cl^−^ ions to balance the total charge of the system and create 0.2 M total salt concentration.

#### Energy minimization

Energy minimization for each structure was performed by using the steepest descent algorithm with an initial step size 0.02 nm. Minimization converged when the maximum force became smaller than 1 kJ mol^−1^ nm^−1^.

#### Free MD simulation

Prior to the free MD simulation, we performed a pressure equilibration in constant temperature and volume (NVT) ensemble with positional restraints applied to all non-hydrogen protein atoms. Subsequent free MD was set in the NPT ensemble (with constant pressure and temperature). The reference temperature of 298 K was maintained by using a Nose-Hoover extended ensemble with the time constant of the temperature fluctuations at equilibrium of 0.4 ps. The pressure was maintained at 1 atm by the Parrinello-Rahman extended-ensemble pressure coupling where the box vectors are subject to an equation of motion, with isotropic pressure coupling with the time constant of 1 ps. Non-bonded interactions were computed by using particle mesh Ewald method with 10 Å real space cut-off for electrostatic interactions and the switching functions between 10 and 12 Å for the van der Waals interactions. The multiple time-step method was employed for the electrostatic forces; the non-bonded interaction list was constructed using a cutoff of 14 Å, updated every 20 steps. The covalent bonds involving hydrogen atoms were constrained using the SHAKE algorithm (with the MD integration step size, 2 fs). Trajectory coordinates were written down every 0.2 ns of simulation.

The resultant trajectories were visualized and analyzed by means of VMD (Visual Molecular Dynamics) software [85].

Structures of all models under investigation after energy minimization are available as Additional files 2 through 7.

### Sequence analysis

The initial sequence search in the RefSeq database of fully sequenced genomes [86] was performed with PSI-BLAST [87] using the horse cytochrome *c* and the human Apaf-1 sequences as queries. Multiple alignments were constructed with Muscle [88]. The logo diagrams were created and visualized with WebLogo [89].

## Reviewers’ comments

We thank the reviewers for their valuable comments and helpful suggestions that helped us improve the manuscript.

### Reviewer’s report 1: Prof. Andrei L. Osterman, Sanford-Burnham Medical Research Institute, La Jolla, California 92037, USA

Reviewer 1: The manuscript by D. Shalaeva et al. “Modeling of interaction between cytochrome c and Apaf-1: bifurcated salt bridges underlying apoptosome assembly” is addressing an intriguing and fundamentally important problem. How the two seemingly unrelated proteins with distinct evolutionary history, molecular functions and compartmentalization recognize each other and form a unique molecular machine of cellular self-distraction? The importance of this recognition and assembly is quite obvious considering devastating potential consequences of imprecision, the untimely cell death or even more dangerous immortality (as in malignant transformation). Remarkably, while numerous experimental studies in this subject area provided rich and diverse data, none of them proposed a sufficiently detailed mechanistic model. In addition to apparent experimental difficulties, this is also due to the general tendency of such (or any other) studies to focus on one particular technology, which often falls short of providing sufficient resolution to effectively address such a complex task. An integrative approach combining dynamic structural modeling with advanced evolutionary analysis allowed the authors of this study to produce plausible and potentially testable hypotheses about atomic-level interactions, a unique electrostatic bar-code driving apoptosome assembly. The choice of both principal technological components of this analysis is perfectly justified by the dynamic nature of the two underlying (albeit very distinct) processes, heterooligomerization of the apoptosome components and their co-evolution. While, the latter aspect is fascinating by itself, the applied co-evolutionary trajectory approach was also particularly instrumental in elucidating the interacting amino acid residues. This was especially helpful for supporting one of the key hypotheses about rather unusual (but not unprecedented) dual electrostatic interactions between lysine residues emerging in eukaryotic cytochromes with adjacent pairs of dicarboxylic amino acid residues in Apaf-1, as well as about their special role in the apoptosome assembly process. Overall, this elegant study provides us with a remarkable example of insightful structural bioinformatic analysis in the postgenomic era. Despite the unavoidably speculative nature of its conclusions, the overall picture is quite compelling and will likely withstand the scrutiny of further experimental studies. Triggering and guiding the follow-up verification experiments, even if potentially refuting some of the conjectures in the work of Shalaeva et al., may be considered one of the key impacts of this publication

*Authors’ response: We thank the reviewer for these interesting comments, and agree that evolutionary studies played the key role in establishing significance of predicted interactions. How the cytochrome c dependent apoptotic mechanism may have emerged is an intriguing question indeed. In organisms with cytochrome* c-*independent apoptosome formation, Apaf-1 molecules are prevented from oligomerization by being bound to some cellular partners and being released only in response to an apoptotic signal, see* [11] *for a review. Most likely, cytochrome* c *got involved in one of such apoptotic cascades simply by chance, providing an additional efficient link between mitochondrial damage and apoptosis. On one hand, the small size of cytochrome c and its location in the intermembrane space lead to its prompt appearance in the cytoplasm after mitochondrial damage. On the other hand, the lysine residues of cytochrome c, which evolved already within bacteria to facilitate the interactions within respiratory chains* [14]*, could complement a number of surface acidic residues of the WD domains of Apaf-1; these residues are generally typical for WD domains* [17, 19, 90, 91]*, which, apparently, also emerged within bacteria* [92]*. Nature could just select for a binding mode for cytochrome c that would lead to the activation of Apaf-1. Further selection would just have increased the specificity of interaction between cytochrome c and Apaf-1.*

*In the revised manuscript, we discuss in more detail the evolutionary implications from our study, as well as the potential verification experiments.*

### Reviewer’s report 2: Prof. Narayanaswamy Srinivasan, Molecular Biophysics Unit, Indian Institute of Science, Bangalore 560 012, India

Reviewer 2: Authors of this manuscript are proposing a three-dimensional model for the complex between cytochrome c and Apaf-1 which contains WD domain. The basis of generation of this model is a strategic integration of extensive sequence, structural and evolutionary analyses with molecular dynamics simulations. Among the multiple models initially arrived at, authors favor one of the models which is consistent with known interaction properties, mutations, conservation of important residues etc. Interestingly the proposed model is radically different from a previously derived model which was determined on the basis of a low-resolution cryoEM map; but, the proposed model too fits quite well in the cryoEM density map as reflected by a good correlation coefficient.

*Authors’ response: We thank the reviewer for the comments. We would not say that our model structure radically differs from the cryo-EM-based model of Yuan and co-workers* [24, 25]*. In fact, we built upon their model, which revealed the location of cytochrome c within the lobe between the two WD domains. Our modeling procedures aimed at refining the orientation of cytochrome c within this lobe.*

Reviewer 2: The approach of the authors is quite effective and the final model seems to fit-in not only in the cryoEM density map, but, also is quite consistent with current understanding of molecular processes in apoptosome. I wish this article is published as it provides an opportunity to those working in this area of apoptosome to consider an alternate effective structural model. However authors may want to consider following points before the possible publication of this work:

Question 1. It is not clear if the flexibilities associated with the tertiary structures of cytochrome *c* and Apaf-1 have been used when authors performed protein-protein docking using various methods. I thought, at some stage in the docking (perhaps at least in the final stages after the interaction patches are recognized), it is appropriate to allow some flexibility in the structures of the two associating interfaces.

*Authors’ response: The software that we used for calculating the protein-protein docking operated with rigid bodies (ZDOCK and PatchDock servers) or incorporated only side-chain flexibility (ClusPro). Therefore, to refine model structures and to examine the flexible interfaces, we have used manual editing and energy minimization procedures and, at the final stage, free molecular dynamics simulations. We have added the respective clarification to the Methods.*

Question 2. When authors fitted their model in the cryoEM density map, have they used flexible fitting? Use of flexible fitting in the density map is likely to result in a better fitting. When flexible fitting is done, are the structural interaction features proposed by the authors remaining undisturbed?

*Authors’ response: We have used a rigid fitting procedure as implemented in Chimera software. It could not be excluded that, if applying flexible fitting, we would end up with a model structure similar to the structure of Yuan and co-workers as shown in Fig.**1**a and b and described in [*25*]; these authors, upon producing their model structure, have used a sophisticated flexible fitting routine complemented by a manual evaluation. Our more modest fitting routine has been applied just to demonstrate that our model structure is compatible with the cryo-EM data.*

Question 3. After authors fitted their model in the cryoEM density map, are there any densities within the zone of cytochrome *c* and Apaf-1 complex in the map that is unoccupied by any part of the proposed model?

*Authors’ response: The arrangement of the WD domains of Apaf-1 in our model structure matched perfectly the arrangement of these domains in the cryo-EM-based model of Yuan et al. [*25*]. However, cytochrome c “sits” more deeply in the PatchDock’ model than in the cryo-EM-based model of Yuan et al. [*25*]. In the latter case, cytochrome c is less deeply buried in the cavity between the two WD domains of Apaf-1, “peeking” slightly out of the estimated electron density (Fig.**1**a and b) and, consequently, leaving a part of the electron density map underneath cytochrome c unoccupied. In contrast, the deeper position of cytochrome c in the PatchDock’ model results in an unoccupied density in the cryoEM map close to the surface of the WD domains (Fig.**1**c and d). In the revised version of the manuscript, we have updated the respective figure by showing the structural models in two projections (see Fig.**1**) to make the difference between the fits of the crystal structures into the electron density map, as obtained in [*24*], for the PatchDock’ model and the cryo-EM based structure [PDB:3J2T] [*25*], respectively, more clear. We also described the differences between the fits in more detail.*

Question 4. What are the calculated energies of interaction between the two proteins in the proposed model and in the model proposed previously?

*Authors’ response: In the revised manuscript, we provide estimates of the changes in solvation energy of the cytochrome c upon its binding to Apaf-1 (ΔG*^*s*^*) for all model structures that were obtained after energy minimization, as well as for the model structure by Yuan et al. [*25*]; the results are presented in the new Table**2**and discussed.*

### Reviewer’s report 3: Dr. Igor N. Berezovsky, Bioinformatics Institute, Agency for Science, Technology and Research (A*STAR), Singapore 138671, and Department of Biological Sciences, National University of Singapore, Singapore, 117597, Singapore

Reviewer 3: The contribution of bifurcated salt bridge to the assembly of apoptosome is hypothesized and explored in this work. Specifically, interactions between cytochrome C and Apaf-1 protein were studied by means of protein-protein docking followed by molecular dynamics simulations. Sequence analysis was used for checking the evolutionary conservation of pairs of acidic residues in Apaf-1 involved in formation of bifurcated salt bridges. The novelty of this research is in unraveling potential role of bifurcated salt bridges in stabilization of the protein-protein interface.

The salt bridge is typically provided by electrostatic interactions and/or hydrogen bonds, depending on the ionization state of relevant residues. The term ‘bifurcated hydrogen bonds’ was first introduced almost 50 years ago [93], the omnipresence of these bonds in proteins was later shown, and geometric characteristics were analyzed in detail [94]. Coincidentally, this reviewer worked on the analysis of hydrogen bonding in protein [58], which revealed substantial role of bifurcated (one acceptor of the proton interacts with two donors) and double (one donor interacts with two acceptors) hydrogen bonds in forming native structures of proteins [59]. Specifically, it appears that about two-thirds of all hydrogen bond in the protein are involved into bifurcated or double bonds (or both). In addition to archetypal backbone hydrogen bonds i-(i + 4) in α-helices, there are also i-(i + 3) hydrogen bonds in about 85 % cases. Overall majority (89 %) of hydrogen bonds in α-helices participate in bifurcated or double bonds. Noteworthy, rigorous geometric criteria used in our analysis [45] delineates all potential hydrogen bonds, which are not necessarily simultaneously present in the protein and vary depending on relevant physiological conditions. MD simulations used by authors allow one to detect dynamic interactions – temporal bonds that can be absent in the crystal structure. While thorough quantitative analysis of the contribution from bifurcated bonds to protein stability remains to be performed, this work unravels another important aspect of these bonds relevant to protein-protein interactions. Pending experimental verification, role of bifurcated bonds in stability of interfaces is a valuable addition to our understanding of the protein-protein interactions and the mechanisms of their formation and stability.

*Authors’ response: We are grateful to the Reviewer for these comments and for providing useful references to the earlier studies of the complex salt bridges/hydrogen bonds in proteins. We have incorporated these references into the revised manuscript.*

*We also appreciate the notion that, according to the current terminology for hydrogen bonding “our” complex salt bridges, where one donor interacts with two acceptors, should be called “double salt bridges” instead of “bifurcated salt bridges”. And still we have retained the designation “bifurcated salt bridges” in the revised manuscript because of the following reasons.*

*First, the term “double salt bridge” has become ambiguous; it is also used to describe a combination of two pairs of residues forming two “parallel”, simple salt bridges next to each other, usually on the interface of two proteins or domains [*95*]. Second, Google Scholar retrieved the first usage of the term “bifurcated bond” as early as in 1941 and in relation to the bonds within a glycine crystal where an amino group of one molecule made a bifurcated bond with carboxyl groups of two neighboring glycine molecules [*46*,*^47^*]. Apparently, this arrangement is exactly the one that we have described for the bifurcated salt bridges in the Apaf-1/cytochrome c complex. In addition, the general theory of hydrogen bonding in solids calls the bonds “bifurcated”, “trifurcated” and “multifurcated” depending on number of proton acceptors interacting with a single donor [*48*].*

*Thus, we decided to keep to the term “bifurcated” as it clearly reflects the main steric feature of the described interactions: a residue of one protein interacts with two residues of the other protein.*

Question 1. Though assignment of the protonation state is described in Methods, it would be important to discuss in the paper what kind of interactions are detected in this case, to compare characteristics of obtained bonds with those typical for ion pairs and hydrogen bonds.

*Authors’ response: For protonation state assignment we have used the PROPKA [*78*] software that is based on empirical approach and not on electrostatics calculations. The desolvation effects, hydrogen bonds and interaction between charges are described by a set of empirical rules, with function formulas and numerical values were “ultimately chosen based on trial and error” [*78*]. Based on an available protein structure and said empirical relationships, this method, from our experience, enables fast and reliable, as compared to experimental data, predictions of pKa values within a couple of seconds. For the Apaf-1 and cytochrome c, PROPKA predicted the lysine residues to be protonated (positively charged) whereas residues of aspartate and glutamate to be deprotonated (negatively charged). Of course, this is not always the case in proteins, and for buried, functionally relevant amino acid residues deviations from this rule were described [*96*]. However, as long as the residues that were implied in the formation of salt bridges between cytochrome c and Apaf-1 were exclusively surface located, these trivial assumptions on their protonation states seem to be reasonable. The pairs of neighboring acidic residues on the surface of Apaf-1 could, in principle, share a proton even in spite of their surface location. However, in the presence of a positively charged lysine residue (see Figs.**2**and**3**) even partial protonation of these carboxyl groups is extremely unlikely because of straightforward electrostatic reasons.*

Question 2. Referring to “dynamic nature” of interactions that can be observed in MD simulations, it would be interesting to analyze Fig. 5 in terms of major states (long-living interactions) existing between corresponding residues.

*Authors’ response: We thank the reviewer for this comment. Indeed, the key feature of the interactions described is their dynamic nature; none of the contacts observed was long-living. Instead, each particular contact was lost and then regained at picoseconds. The only exceptions were salt bridges between residues Lys25 and Asp941 as well as Lys8 and Asp1147, which could be maintained for up to 10 ns, see Fig.**5**. In the revised manuscript, we have updated Fig.**5**to include the graph for distance between Lys86 and Asp1064, and have rescaled the Y axis (distances) to better illustrate the mobility of residues. To provide further information about the dynamic properties of the salt bridges, we have added a new Table**3**into the revised manuscript. In addition, we plotted the distances between proton donor and acceptor atoms of interacting residues against each other for each of the three stable bifurcated bridges (see the new Fig.**6**).*

Question 3. The binding of cytochrome C to WD domains of the apoptotic activating factor Apaf-1 is generalized/hypothesized in the discussion onto the potential role of WD domains in “transmitting mechanical signals rather than their purely structural role”. This idea should be explained and formulated in more clear way.

*Authors’ response: We have expanded the respective section of the Discussion.*

### Reviewer’s report 4: Prof. Gerrit Vriend, Centre for Molecular and Biomolecular Informatics, Radboud University Medical Centre, Nijmegen, The Netherlands

Reviewer 4: I am not familiar with cytochrome c at all and poorly read-in on apoptosis, which, I guess, disqualifies me a bit as a referee. But I will do my best.

1. As a bioinformatician, I generally get worried when I read that protein structures got ‘improved’ by molecular dynamics. MD is a nice technique, but our YASARA experiences [85] made clear that MD normally drives structure models away from the true minimum.

*Authors’ response: We fully agree with the notion that MD simulations might drive structures away from the true energy minima. Therefore, in our article, we first obtained energy minimized model structures and only then used MD simulations to tackle the dynamics of some of them. In the revised version we have replaced ‘improved’ with a more appropriate wording.*

2) Along the same lines as point 1, I am worried about salt-bridges by lysines, especially bifurcated ones. Lysine is the residue with the most flexible side chain, so one would expect it to move around liberally enjoying its entropy, while passing by its negatively charged friends frequently. The chance that all of them actually form a salt bridge at the same time seems rather small.

*Authors’ response: We fully agree with this comment. Lysine-involving salt bridges, particularly at the interfaces, are known to be highly mobile and could be present only temporarily ([*42*] and references therein). The mobility of lysine residues follows also from our MD simulations. While the original manuscript already contained a figure (Fig.**7**in the revised version) that demonstrated the mobility of lysine residues, we have now added a new Table**3**with quantitative estimates. .*

3) I doubt that the extra strength of bifurcated salt bridges over ‘normal’ ones is big, but if it is then it is probably not the disturbance of their local backbones that triggers a large conformational movement. The latter is more likely caused by the combination of forces providing something resembling a torque.

*Authors’ response: We thank the reviewer for this comment. The investigation of the mechanics of conformational changes within Apaf-1 upon cytochrome c binding is currently in progress, and we will definitely consider the possibility suggested by the Reviewer.*

4) “..programmed cell death underlying numerous processes..” underlying - > involved in.

*This has been changed.*

5) Nothing to do with this article, but in the SwissProt file for Apad-1/human each WD motif I click on does not point to a stretch that contains a W. I see W…D motifs, though.

*Authors’ response: The terminology for the motif is, unfortunately ambiguous. WD domains are comprised of WD repeats (also called WD40 repeats). These are 40-aa motifs that often, but not always, terminate with a Trp-Asp dipeptide. In the revised manuscript, we avoided using the term “WD motif” and use “WD-repeat”.*

7) and in that same paragraph it would read more logically if the order of the two sentences was reversed.

*Authors’ response: We have deleted one of the sentences.*

8) “..An improved model of the human apoptosome was recently presented by Yuan..” improved over what?

*Authors’ response: The model of the human apoptosome presented by Yuan et al. in 2013* [25] *was an improvement over the cryo-EM structure with a 9.5 Å resolution, which had been obtained by the same team three years earlier* [24]*. In the’improved’ “structure, cryo-EM data were combined with crystal structures of respective proteins* [25]*. We have modified the wording to clarify this point.*

9) “..have identified several residues of cytochrome *c* that seemed..” they either have been identified as involved, or they seem involved, but not both options at the same time.

*Corrected.*

10) “..used to measure the caspase-9 activation in presence..” - > used to measure caspase-9 activation in the presence.

*Corrected.*

11) “..the numbering matches the mature horse and human cytochrome c sequences without the N-terminal methionine..” it would help to add here which PDB or SwissProt files correspond to these two molecules.

*Done*.

12) “.. The only non-lysine residue mutations with a similar effect were the Glu62Asn replacement in horse..” it would be nice to know how many non-lysine residue mutations were tried.

*Authors’ response: Since there are numerous lysine residues on the surface of cytochrome c, these residues were expected to be involved in the interaction with aspartate-rich WD domains and mutational studies were mostly focused on lysine residues. There were some non-lysine residue mutations, mainly in the paper by Yu et al.* [29]*, which includes mutations of 13 different non-lysine residues that had no significant effect on apoptosome assembly. We have included the information of the total number of non-lysine residues being mutated into the revised manuscript.*

13) “.. Kokhan and colleagues found that many dynamic hydrogen bonds and salt bridges, transiently showing up in MD simulations [42], were absent from the available high-resolution crystal structures..” This can, of course, be interpreted in several ways; for example, one can assume that MD moves things closer to the energy minimum in its own force field space, or just the other way around, that the static X-ray structure, with all its crystallization artefacts, failed to reveal these interactions.

*Authors’ response: Kokhan and colleagues in their work refer to the static nature of crystallographic structures unable to reflect mobile interactions. We have added their interpretation to the revised manuscript.*

14) And about the next line: could simple, Delphi-style electrostatic complementarity calculations not have found the same lysine – negative residue proximities?

*Authors’ response: This depends on the criteria used for detecting interactions, and what distances exactly are defined as “proximities”. Undoubtedly, one can see the possibility of interaction between two residues located close enough to each other. However, the possibility of said interaction is also affected by the space of possible rotamers for each residue, interactions with other residues and non-protein molecules, etc. We are not aware whether Kokhan et al. used Delphi-style electrostatic complementarity calculations. In principle, MD simulations are sensitive to electrostatic interactions and enable not just their detection, but also estimation of their probability in form, for instance, of the contact time between two partners during the simulation (see Fig.**5**).*

15) “..We analyzed the interaction between cytochrome c and the WD domains..” repeated too often.

*Removed on a few occasions.*

16) “.. Since Apaf-1 surface is enriched with” surface - > the surface.

*Corrected.*

17) “..that this interaction is specific and requires not just a positively charged..” would be nice to know how many positive and negative charges are involved, and what the charge complementarity looks like.

*Authors’ response (17): We have expanded and modified Fig.**4**to show the electrostatic complementarity between the interacting surfaces of cytochrome c and Apaf-1.*

18) Was there a reason to not also include results from the nowadays rather popular HADOCK software?

*Authors’ response: We thank the Reviewer for the suggestion and we will consider using this software for future studies. With the wide range of software for protein-protein docking available nowadays, it was impossible to use all. We have chosen the programs PatchDock, ZDOCK and ClusPro, as they include a “fast and rough” approach (PatchDock), “slow and sofisticated” (ClusPro, which apparently is similar to HADOCK) and a compromise between the two (ZDOCK).*

19) “..and provide as many lysine-aspartate/glutamate pairs as possible..” what is really meant with this? If all four models had already the maximal possible number of salt bridges, then they must all four be rather similar, and MD optimization would not achieve much extra.

*As documented in the manuscript (Table**1**and Additional files), the three structures that were obtained by different docking software tools were quite distinct. They provided different salt bridges and also the numbers of salt bridges were different. Furthermore, in the case of the PatchDock’ structure the number of salt bridges increased dramatically after energy minimization (Table**1**). The Reviewer is quite right that application of the MD routine did not increase the number of salt bridges any further.*

20) “..manual adjustment yielded..” always worries me a bit and might need a bit more justification.
21) “.. Therefore, during manual editing, we adjusted the position of this loop in all model structures to provide salt bridge partners..” how was this done?

*Authors’ response: During manual editing and further evaluation of model structures we used the presence of salt bridges including functionally important (as shown by experiments) residues as the main criteria. Thus, during manual editing we have adjusted the amino acid positions, if such an adjustment yielded a new salt bridge and did not require significant disturbance of the structure. In one case, we succeeded to slightly tilt the whole molecule of cytochrome c, providing salt bridge partners for the four functionally most important lysine residues (the PatchDock’ structure).*

*The difference between the model structures, as provided by different docking routines, might be, to some extent, specific to the interaction studied. Indeed, the small globule of cytochrome c is almost evenly and densely covered by 18 lysine residues; almost each of them can potentially make a salt bridge with acidic residue (s) of a WD domain. In the revised manuscript, we explicitly state that although our model structure may be a non-unique solution as it concerns the orientation of cytochrome c, this model structure enabled the identification of the three acidic duplets of Apaf-1 that, on one hand, are involved in complex, bifurcated bonds with the lysine residues of cytochrome c and, on the other hand, show a distinct evolutionary pattern, appearing only within Chordata, concomitantly with the appearance of the cytochrome c-dependent apoptotic pathway. Since only three acidic duplets of Apaf-1 are in a position to interact with cytochrome c (see Figs.**4**and**10**), we believe that these acidic pairs might bind cytochrome c, thus triggering the apoptosome formation.*

22) “..and in each of these models, lysine residues of cytochrome *c* formed several salt bridges..” how many lysines did this, all of them? Quantify, please.

*Authors’ response: A list of all lysine-involving salt bridges for each model, calculated before and after energy minimization, is presented in Table**1**.*

23) “.. Notably, the ClusPro model changed insignificantly after energy minimization, while the manually edited PatchDock’ model gained 6 new salt bridges..” this probably is the result of one docking server using EM/MD and the other not, or both using different force fields, one of which is similar to yours?

*Authors’ response: The ClusPro server used a MD approach with the CHARMM force field, same as we used in the MD simulations, so the consistency of energy minimization results was expected. The other two docking programs did not incorporate energy minimization procedures. The PatchDock’ model was the most perturbed, as compared to the outcome of the docking routine, because of the manual editing, which might explain the pronounced effect of energy minimization.*

24) I don’t think 45 ns is a long enough simulation to say anything about stability of the whole complex, especially given the enormous size of this complex.
25) “.. Thus, MD simulations revealed only one model (the PatchDock’ model, Fig. 1) that kept the proper domain architecture and intact geometry during the MD simulation..” this worries me. Could it be that a much more careful equilibration of MD is needed? Or that the complexes are wrong?

*Authors’ response: As we have explicitly emphasized in the revised manuscript, the model structures might be all wrong, they are just theoretical predictions that await experimental scrutiny. Our task was, however, to identify the residues of Apaf-1 that are involved in binding of cytochrome* c*. We believe that we have solved this problem by combining structural modeling with sequence analysis.*

*We had to limit our MD simulation time to 45 ns due to the large size of the system. Still, we think that the simulation time was sufficient to discriminate a mechanically “wrong” structure from a stable one.*

*The heat maps in Additional file**1**: Figure S1 show that while the stability of the ClusPro structure decreased with time, the stability of the PatchDock’ structure increased through the MD simulation. So it seems unlikely that the PatchDoc’ structure would break up upon a longer MD simulation.*

26) “..of Apaf-1 is more or less evenly negatively charged..” more or less?

*Deleted*

27) “..correlation coefficient of 0.9463 as compared to 0.9558..” how calculated?

*Authors’ response: We have used UCSF Chimera package* [84]*. The reference to this software has been added to the Methods section.*

28) * Error:* “.. Electrostatic/polar interactions or bonds that include salt bridges and potential H-bonds are generally considered within a 4 Å cutoff..” the 4A cutoff is for H-bonds. Salt bridges tend to have a cutoff of 8-12A or even longer. The shorter salt bridges sometimes are called H-bonded salt bridges. This also why there should be at least 12A between the solute and the simulation box…

*Authors’ response: We do not see an error here. The criterion for identifying a salt bridge, as originally proposed by Barlow and Thornton* [54]*, is that the distance between the heavy atoms of the ionizable groups of charged residues should be less than 4 Å. This cut-off of 4 Å has been used for defining salt bridges in numerous studies, see* [50–53] *and references therein, as well as in the previous studies of cytochrome* c *interactions with its partners* [42]*. The cut-off of 4 Å was also taken for salt bridges in the paper of de Groot and co-workers* [49] *that was co-authored by the Reviewer. We have added the references to all these classical papers to the revised manuscript. It is important to note that we also discuss the long-range interactions. In the original manuscript, we have considered a cut-off of 5 Å, as experimental studies show detectable interactions even at this distance* [55]*, in addition to the 3 Å cut-off used to identify strong hydrogen bonds (Table*3*in the revised manuscript). To address this comment of the Reviewer, in the revised manuscript, we have added the data that had been collected with a cut-off of 6 Å to illustrate that any further increase in the cut-off length is “washing out” the differences in the population of salt bridges.*

*The ‘cutoff of 8-12A or even longer’ mentioned by the Reviewer, might be related not to salt bridges per se but to “longer range ion pairs” (as defined by Nussinov and co-workers, see* [50, 51]*). We were not interested in such weak interactions since they were unlikely to contribute to triggering a major rearrangement of the WD-7 domain of Apaf-1 upon the binding of cytochrome c.*

*As for electrostatics interactions in general, for MD simulations we used a 10 Å cut-off for coulombic interactions and 14 Å cut-off for all long-distance interactions with combination of PME and a switch function for the direct-space part.*

29) The story about “..angle Θ between the Cα atoms..” is better left out. It weakens the story. There is no sensible justification for this that I can think of that doesn’t automatically goes with the wash in MD.

*Authors’ response: We would rather leave this part in since the cooperativity of the complex salt bridges, which is determined not by the exact nature of the lysine residue, but by the neighboring position of the two aspartate residues, might be important for triggering the rearrangement of Apaf-1..*

30) Any sentence that starts with “..As already noted..” can be deleted. Here too.

*We would rather keep it as it is a reference to prior work.*

31) If lysines increase (evolutionary) at the one side of the binding interface, then what about the negative charges at the other side?

*Authors’ response: We now address this point in the second part of the’Sequence analysis’ section and in the Discussion section of the revised manuscript.*

32) The discussion is too much a repeat of the previous, and not enough a discussion.

*Authors’ response: In the revised manuscript, we deleted the repeats (at least, some) and have substantially expanded the Discussion.*

33) In Fig. 3 I would have loved to see how well the electrostatic potentials around the two proteins that are docked fit, or how well things cancel out, or something like that. After all, nature wants things to be neutral.

*Authors’ response: We have modified Fig.**3**(Fig.**4**in the revised manuscript) to illustrate the electrostatic complementarity.*

34) Is Fig. 4 really needed?

*Authors’ response: Figure**4**is now the Figure**1**of the revised manuscript. It is a comparison of the PatchDock’ model (this work) with the previously published model structure by Yuan et al. [PDB:3J2T]* [25]*. Both models are fitted into experimental cryo-EM density map* [24]*. We think that this figure is useful, as it illustrates that the proposed PatchDock’ model matches the cryo-EM data.*

35) Figures 8 and 9 nicely indicate the sequence patterns, but there is so much distraction that they almost make it harder rather than easier to see things.

*Authors’ response: We used the Sequence Logo representation [*89*], a popular tool for illustrating multiple alignments of large numbers of sequences, for these figures (Figs.**9**and**10**in the revised manuscript). In a such presentation, the statistical significance in each position is cseen. In the revised manuscript, we also add a multiple alignment of the WD domains as Additional file**1**: Figure S2.*

In summary, I think this is a simple study that mainly got complicated by the enormous size of the complex at hand. I indicated one error that should be fixed. I would love to see how their final model fits in the EM density, and I miss a bit the experimental validation, or at least a pointer towards how this should be done.

*Authors’ response: We are happy to see that Reviewer appreciated the scale of the problem that the object of this study has set for theoretical calculations. We thank the reviewer for his very helpful comments. We agreed and have taken into account all of them with the single exception of the one that had been marked as an error by the Reviewer. We still believe that we have used a proper criterion for the salt bridges in our analysis.*

*Figure**1**a and b, the necessity of which has been questioned by the Reviewer in the comment (34), show how our final model fits in the EM density. In the revised manuscript we offer some hints on how the functional consequences from our model might be validated by mutating the acidic residues of Apaf-1. Of course, we hope to see a well-resolved crystal and/or cryo-EM structure of the cytochrome c/Apaf-1 complex in the near future.*

## Additional files

Additional file 1: Figures S1 and S2.
**Figure S1.** Backbone coordinates RMSD heat maps for WD domains of Apaf-1 in complex with cytochrome *c* during MD simulation. **Figure S2.** Conservation of negatively charged residues in the WD domains of Apaf-1 homologs. Additional file 2:
**The PatchDock’ model structure after energy minimization.** This is the structure obtained after manual editing of PatchDock-predicted model and energy minimization. The PatchDock’ model shows the most number of salt bridges involving functionally relevant cytochrome *c* residues and remained stable during molecular dynamics simulations. Additional file 3:
**Original EM-fitted model structure [PDB:3J2T] [** 25 **] after energy minimization.**
Additional file 4:
**The ClusPro-predicted model structure after energy minimization.**
Additional file 5:
**The PatchDock-predicted model structure after energy minimization.**
Additional file 6:
**The first ZDOCK-predicted model structure after energy minimization.**
Additional file 7:
**The second ZDOCK-predicted model structure after energy minimization.**

## Abbreviations

Apaf-1: Apoptotic protease activating factor 1
CARD: Caspase activation and recruitment domain
Cryo-EM: Cryo-electron microscopy
ETC.: Electron-transfer chain
MD: Molecular dynamics
NBD: Nucleotide-binding domain
ROS: Reactive oxygen species
