# Supplementary material for: Modeling of interaction between cytochrome c and the WD domains of Apaf-1: bifurcated salt bridges underlying apoptosome assembly
Source: Biol Direct. 2015 May 27;10:29. doi: 10.1186/s13062-015-0059-4 (PMC4445527; doi:10.1186/s13062-015-0059-4)
Supplement: Additional file 1: Figures S1 and S2. — Figure S1. Backbone coordinates RMSD heat maps for WD domains of Apaf-1 in complex with cytochrome c during MD simulation. Figure S2. Conservation of negatively charged residues in the WD domains of Apaf-1 homologs. [file 13062_2015_59_MOESM1_ESM.pdf]

Supplementary material

to the paper by D.N. Shalaeva, D.V. Dibrova, M.Y. Galperin, and A.Y. Mulkidjanian

"Modeling of interaction between cytochrome c and the WD-domains of Apaf-1: bifurcated salt bridges underlying apoptosome assembly"

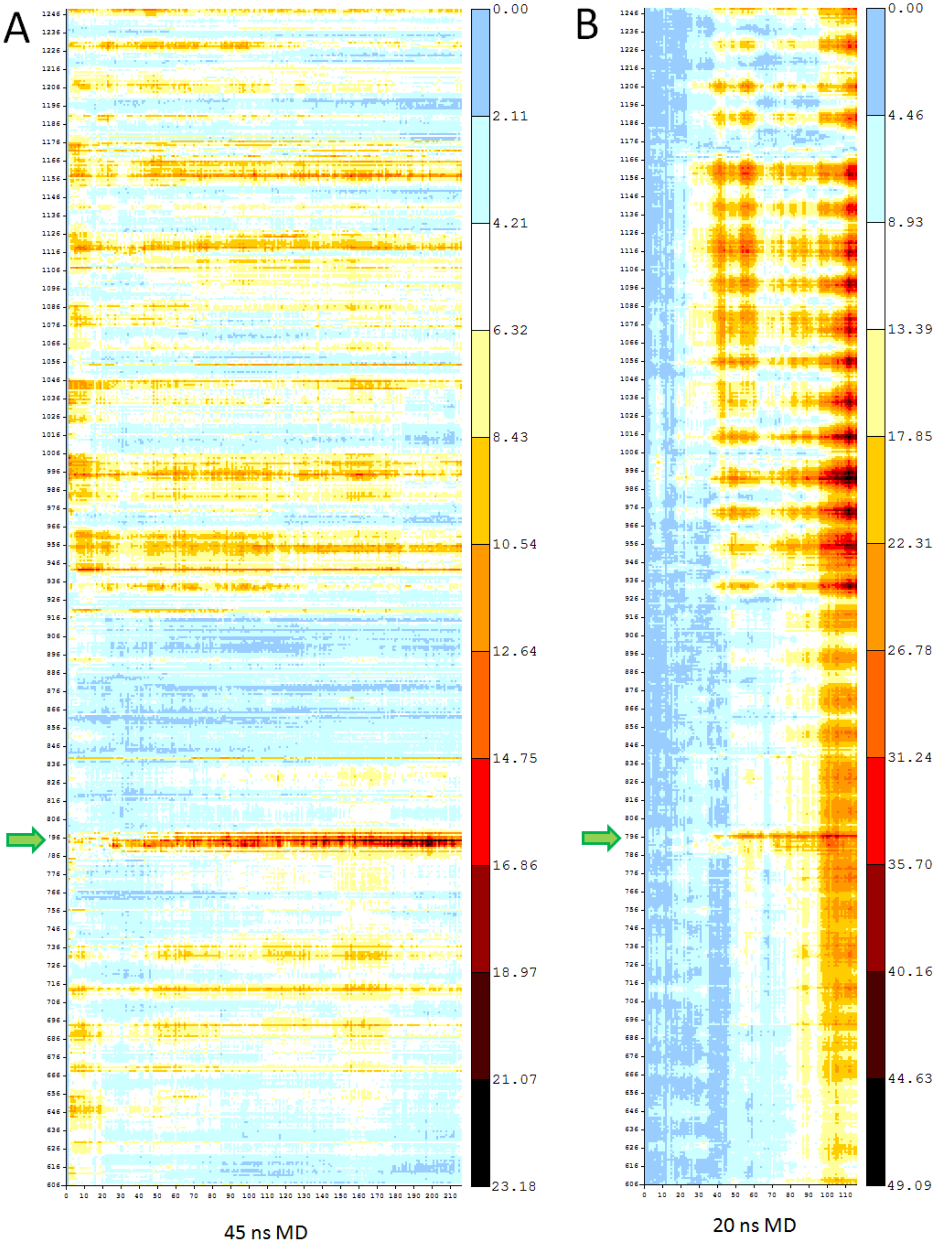

Figure S1. Backbone coordinates RMSD heat maps for WD domains of Apaf-1 in complex with cytochrome *c* during MD simulation. A, The final model structure (model PatchDock'); B, ClusPro predicted structure. The green arrow indicates residues 785-805 that form a mobile loop. Created with VMD tools.

```
APAF_HUMAN     612  RPHTDAVYHACFSEDGQRIASCGADKTLQVFKAETGEKLLEIKAHEDEVLCCA-FSTDDRFIATCSVDKKVKIWNSMTGELVHT-YDEHS  699
APAF_MOUSE     612  RPHTDAVYHACFSQDGQRIASCGADKTLQVFKAETGEKLLDIKAHEDEVLCCA-FSSDDSYIATCSADKKVKIWDSATGKLVHT-YDEHS  699
APAF_DANRE     614  HPHQGAVYYACFSKDGSKIASCGASKALRVFKSTSGEKLLELQAHEEDVLCCA-FSPDDRHIATCASDRKVKLWNVERGVLIREFEVEHE  702
XP_011682983   616  TPHSGVTMCAQFNAAGDHVLSCGEDGYVKFWSSESGKQAFSIEAHNNWVNWCE-ISPDGQTMVTCSS-SNVKLWRIPNGGYPLHQAMIRK  703
H2DL11_SCHMD   825  ---------------------------------------------------------DDLFCAARNNIYKNR-------ELILT-IE---  846
XP_001639696   604  KVHCGAVYCCKFSSDSSKVVSCGTDNHVKVWDSQSGRQLLSISGHGDVVNCCA-FSHDDSRIISCSADQTVKIWDASSGEGVLC-FVGHT  691
XP_002117644   610  RIHKASAACCSFSPDGSQVVSSSWNGDIQIWDAFSGASVAKFDGHGDEVVCCCCFSKDGR-IASASLDETVKVWNIANESLELV-YDKHD  697
XP_002167203   609  KLHGDAVNYCSLSHNTTLAVSCSRDGMVKVWDYLTGAEKISFSGHDNDVKWCE-FSFDDENIISCSTDKTIKVWNMRSSEEEIC-LRGHT  696

APAF_HUMAN     700  EQVNCCHFTNSSHHLLLATGSSDC--FLKLWDLNQKECRNTMFGHTNSVNHCRFSPDDKLLASCSADGTLKLWDATSANERKSINVKQFF  787
APAF_MOUSE     700  EQVNCCHFTNKSNHLLLATGSNDF--FLKLWDLNQKECRNTMFGHTNSVNHCRFSPDDELLASCSADGTLRLWDVRSANERKSINVKRFF  787
APAF_DANRE     703  EQINHCQFTNTGRRVLLATCSNDKFTNTRLWNPNKKTSQNTMFGHMEPVNHCCFSPNDLYLATSSSDGSLKLFEVSSANEWKSIDVDSFF  792
XP_011682983   704  IPGDWLRAIFSHDGTKIAMCSLDG--LIEILDLTEDRRKMIRASDSVIRALC-YTLDDRYIISCSDDTKVKVRRDRDTRVFDASS-----  785
H2DL11_SCHMD   847  NDVYIVKISISDNQTLLALCLTNN--SVQVWNYYNQTLLYSLNDSQFTDVY--FLHDSDFLLTTGP-TSAAKWDLKNISLANNNNNNSE-  930
XP_001639696   692  QEVFSCSFSPDDTKAV--SCSADR--TVKVWDSKTGVCYHVYMAHTDIVRWCCFSPDGGKVASCSDDNTVRIWEASSGEDL---------  768
XP_002117644   698  ADVLWCQFSPDQANIV--SCGADR--LVKLWNSRTGEDYATFYGHLDIVRQCAFSNDGQKIVSCSDDTYVKVWDTQSGDIRKDLL-----  778
XP_002167203   697  DEVLCCRYSKNKKWIA--STSMDK--SLKIWEGISGKLYISFNDHTDVVNCCSFSNCSNLVVSCSNDCTTRVWNIKEKN-----------  771

                        792
APAF_HUMAN     788  LNLEDPQEDME--VIVKCCSWSADGARIMVAAKNKIFLFDIHTSGLLGEIHTGHHSTIQYCDFSPQNHLAVVALSQYCVELWNTDSRSKV  875
APAF_MOUSE     788  LSSEDPPEDVE--VIVKCCSWSADGDKIIVAAKNKVLLFDIHTSGLLAEIHTGHHSTIQYCDFSPYDHLAVIALSQYCVELWNIDSRLKV  875
APAF_DANRE     793  PESDE---EIK--AMVKCSTWSADGSQIICAARNTVFVFDVETSDLLLKLKTSRLSTIQFCHACPNSSLLAVALSHYTVELWNFESSKKK  877
XP_011682983   786  AMSDPVMSYTGHTTSVINASPSPDGQYVATTGGSDVHVWTIVDGALVGKCMS-EANMVLCCEFCPKGDIIAGGLSSYEIVLWETRRYKPV  874
H2DL11_SCHMD   931  LNSNSLSLYQGHFGMQNQ---SQVSFLLVLQSVNNNIEFFSYTNEILNSKVIKESSEMSCFYFY-ENIIFIGFKRLESIKVYDLTTYEEI 1016
XP_001639696   769  CLLDDHT------SSVSFCCFMNQGESVVTVCSNCIKVWSC-SSGEQTWVYNS-SSTCLSLAFSPDDSIVAMGFSDTTVQLWNSKTFARL  850
XP_002117644   779  LALTHHDE-----GVSSCAFFNNDTMIISSSGRNVITSSVKDGSKLASFRCF---GGVLCCNVSSSGLYFAAGLTTSALQMWHIQDRQTV  860
XP_002167203   772  MTLVCSSG-----DFCTSCIFSLDDQFIFTSCQRFVLKCSSKDGDLLEKIETA--SDILSMALSYNGKHMGLSHQNMCASVMDLSKSEVF  854

                    877                           903                    925
APAF_HUMAN     876  ADCRGHLSWVHGVMFSPDGSSFLTS---SDDQTIRLWETKKVC-KNSAVMLKQEVDVVFQENEV---MVLAVDHIRRLQLINGRTGQIDY  958
APAF_MOUSE     876  ADCRGHLSWVHGVMFSPDGSSFLTA---SDDQTIRVWETKKVC-KNSAIVLKQEIDVVFQENET---MVLAVDNIRGLQLIAGKTGQIDY  958
APAF_DANRE     878  AECSGHLSWVHCVQFSPDGSLLLSS---SDDQTIRLWETDRVH-TSSAVALKRDTDVLSSHSDA---TIIAPDSSNRLQVLSGSTGAVVL  960
XP_011682983   875  AVFKGHTSWVMSVKFDVKGERLVSA---SDDETVRTWKVEYGH-TDLNSVWKRDFTACFCDGDKRLKFVVAGDNKNRCLVTDGRNVDQTF  960
H2DL11_SCHMD  1017  TKISDFVDVQEIYAHKTKTKNILEILVHHSRNKIRILHL--------------------------------------------NQVSL-- 1060
XP_001639696   851  GVYKGHKGWAHSVGISKDSSKLVSG---SEDETVKIWTIDRDA-AKDAAKLRRVFDAHFGEDSL---GVAVADSSDRLLIFQGQDGVLTA  933
XP_002117644   861  AVYKGHNGWIQSVSYAPDDSRLLSS---ASDETVKFWSSNPTEDKFARVKLRIFFTCSFQDDEP---TIYAPDMQESLLVYHGLNSKFVE  944
XP_002167203   855  NNYYGHSSWVNSINFSRDGSRIITA---SADGACKVWSVKDNM-DVEEKLIPV-FDVSFESGFY---IVVGTNQ-KRIKVINECNYAHSA  935

                                              981                                     1023
APAF_HUMAN     959  LTEAQVS---CCCLSPHLQYIAFGDENGAIEILELVNNRIFQSRFQHKKTVWHIQFTADEKTLI-SSSDDAEIQVWNWQLDKCIF-LRGH 1043
APAF_MOUSE     959  LPEAQVS---CCCLSPHLEYVAFGDEDGAIKIIELPNNRVFSSGVGHKKAVRHIQFTADGKTLI-SSSEDSVIQVWNWQTGDYVF-LQAH 1043
APAF_DANRE     961  ESEELSSRIRCSCISRNAAFVALGSEDGTVQVIEVPSSKASVKLSGHTKTVHHCQFTDDCEILI-TSSEDSTIRVWKWRTGECMV-LQGH 1048
XP_011682983   961  VVSEESRRIRAFALTSDNTKVAYGSDGGVVKVANVETHAVERSLEAHTMDVRQCLFTKEGDTLV-TCSSDGLIKIWPEG-GEVIE-CRGH 1047
H2DL11_SCHMD  1061  ------------------------------------LKVKDSTCELPGNSINCKFFKYEGDIVKFVSICDIQCYLCDVNEVIKPIHTYNL 1114
XP_001639696   934  EGEAMDSKIHSVTFSPRGEAIAVGCESGTISIQDR-SCKVLQSFSEHKGAVRRCYFSQDGQLLL-SASDDKTAKVFNLQEARVMYTLAGH 1021
XP_002117644   945  EYSEGRSKIRCCCLSKDETLLSSGRENGEVMILSSIDGRLLQELNGHKDTIIFIRFIGDRKRLL-SCARDNTAKVFNENMTVSMY----- 1028
XP_002167203   936  SSLQEQFLDLSWGHP---------------------------------------------------------------------------  950
```

```
                      1045
APAF_HUMAN   1044  QETVKDFRLLKNSR---LLSWSFD----GTVKVWNIITGNK--EKDFVCHQGTVLSC----DISHDATKF-SSTSADKTAKIWSFD--LL  1117
APAF_MOUSE   1044  QETVKDFRLLQDSR---LLSWSFD----GTVKVWNVITGRI--ERDFTCHQGTVLSC----AISSDATKF-SSTSADKTAKIWSFD--LL  1117
APAF_DANRE   1049  MEPVRKFHLLSSSSSPHLFSWSFD----GTVKVWDLTRGQM--LQDLVCHEGAVLSC----DVSSDGRLF-ATTSANRTAKVWSSA--SW  1125
XP_011682983 1048  TKAVTQCQLYQNDAK--ILSSSRD----ATLKIWELSTGHC--LATIEAHSDWIFMC----AISPDHSMV-ASVSVDKTAKVWDLQ--SH  1122
H2DL11_SCHMD 1115  ENNVLDVDIDDNCQY--LCAISNN----GEIKVWEIGTELSCVNEEESLIGNILRKCKFRPKMSPNNWNVVAVINDNAIISLFYFRGIEQ  1198
XP_001639696 1022  TNKLRRCMFFNNDTR--ILTASLD----GSLKVWDAKTGNL--EFT-CCHSSTDYVLTC--DVSHDSQRL-ISASADCFAKVWDAS--TG  1097
XP_002117644 1029  ---KIDRLLQLFVAK--VILYSFVYMHLLLGILWDSATGNM--LYD-YSHSGHLTDC----SIMPDQQSF-ITTSVDAIAKLWSCE--DG  1103
XP_002167203  950  ------------------------------------------------------------------------------------------   950

                                                  1147
APAF_HUMAN   1118  LPLHELRGHNGCV----RCSAFSVDSTLLATGDDNGEIRIWNVSNGELLHLCAPLS-EEGAATHGGWVTDLCFSPDGKMLISAGGYIKWW  1202
APAF_MOUSE   1118  SPLHELKGHNGCV----RCSAFSLDGILLATGDDNGEIRIWNVSDGQLLHSCAPISVEEGTATHGGWVTDVCFSPDSKTLVSAGGYLKWW  1203
APAF_DANRE   1126  KMLFLLEGHKDCV----RSCRFSWDNKRLATGDDNGEIRLWSMLDGALLKICPRDTKDSMNSYHAGWVTDLHFSPDNRVLVSTAGYIKWW  1211
XP_011682983 1123  KLRKVLDNHIDSV----RTCRFSPDSTLLATGDDSGIVRIWNISSGDEVGEC---------HKHKSWVTDIKFSPDAKIVMTAGENVKWW  1199
H2DL11_SCHMD 1199  KLKILIDPYETVATQIPNFIKWSIDGNYVLVGDYN--IRKWKVPPEPLKKDE---------NKLPLTILQTYF-----------------  1260
XP_001639696 1098  ELLLSLGRHPDVV----RSISFSPDNMI-CTGCDDGIVRIWDSVSGKEVTSCKK---------NETWIADCHFTRDGRNIVSVSDNIQWW  1173
XP_002117644 1104  RLIRSYTAHEDCV----RSCRISNDGKLLAVGCDNGSIKIWDIHSGKELMTL---------RGHNVWVNCCKFSKDGNYLVTVSDTLIWW  1180
XP_002167203  950  ------------------------------------------------------------------------------------------   950

APAF_HUMAN   1203  NVVTGESSQTFYTNGTNLKKIHVSPDFKTYVTVDNLGILYILQTLE  1248
APAF_MOUSE   1204  NVATGDSSQTFYTNGTNLKKIHVSPDFRTYVTVDNLGILYILQVLE  1249
APAF_DANRE   1212  SVESGEALQTFYTMGGNLKKIHVSPDFSTFITVDSIGILYILKRLE  1257
XP_011682983 1200  Q-LDGTLLQTFHVRGTFIRQVQASPSFQTFVTIDSAGILYLMCRVK  1244
H2DL11_SCHMD 1261  -----------------GGIQNISLNEKFICTVDQQGFFYILKKID  1289
XP_001639696 1174  S-KSGQLIQEFFIKGGFAKDVKCSSDFKTFVTVDDTSTLYILNQI-  1217
XP_002117644 1181  DLRNGKKLQEIRLFGRYADDIFFNGDFTHFVTIDSESIVYILKRLT  1226
XP_002167203  950  ----------------------------------------------   950
```

Figure S2. Conservation of the negatively charged residues in the WD domains of Apaf-1 homologs.
Multiple alignment of the WD domains of Apaf-1 homologs from human (APAF_HUMAN, RefSeq accession NP_863651), mouse (APAF_MOUSE, NP_033814), zebrafish (APAF_DANRE, NP_571683), sea urchin *Strongylocentrotus purpuratus* **(**XP_011682983), planarian flatworm *Schmidtea mediterranea* (H2DL11_SCHMD, no RefSeq entry), sea anemone *Nematostella vectensis* (A7RLM8_NEMVE, XP_001639696), placozoan *Trichoplax adhaerens* (B3SBJ1_TRIAD, XP_002117644), and the hydrozoan *Hydra vulgaris* (XP_002167203) was generated from BLAST outputs. Conserved acidic residues are shown in bold red font. The residues that are involved in cytochrome *c* binding in the PatchDock' model (Table 3) are shaded yellow and their positions in human Apaf-1 are indicated above those residues.
